# Basal forebrain innervation of the amygdala: an anatomical and computational exploration

**DOI:** 10.1007/s00429-024-02886-1

**Published:** 2025-01-13

**Authors:** Tuğçe Tuna, Tyler Banks, Gregory Glickert, Cem Sevinc, Satish S. Nair, Gunes Unal

**Affiliations:** 1 Behavioral Neuroscience Laboratory, Department of Psychology, Boğaziçi University, Bebek, 34342 Istanbul, Turkey; 2 Neural Engineering Laboratory, Department of Electrical Engineering and Computer Science, University of Missouri, Columbia, MO 65211 USA

**Keywords:** Basal forebrain, Amygdala, Cholinergic, GABAergic, Computational model, Oscillation

## Abstract

Theta oscillations of the mammalian amygdala are associated with processing, encoding and retrieval of aversive memories. In the hippocampus, the power of the network theta oscillation is modulated by basal forebrain (BF) GABAergic projections. Here, we combine anatomical and computational approaches to investigate if similar BF projections to the amygdaloid complex provide an analogous modulation of local network activity. We used retrograde tracing with fluorescent immunohistochemistry to identify cholinergic and non-cholinergic parvalbumin- or calbindin-immunoreactive BF neuronal subgroups targeting the input (lateral and basolateral nuclei) and output (central nucleus and the central bed nucleus of the stria terminalis) regions of the amygdaloid complex. We observed a dense non-cholinergic, putative GABAergic projection from the ventral pallidum (VP) and the substantia innominata (SI) to the basolateral amygdala (BLA). The VP/SI axonal projections to the BLA were confirmed using viral anterograde tracing and transsynaptic labeling. We tested the potential function of this VP/SI-BLA pathway in a 1000-cell biophysically realistic network model, which incorporated principal neurons and three major interneuron groups of the BLA, together with extrinsic glutamatergic, cholinergic, and VP/SI GABAergic inputs. We observed in silico that theta-modulation of VP/SI GABAergic projections enhanced theta oscillations in the BLA via their selective innervation of the parvalbumin-expressing local interneurons. Ablation of parvalbumin-, but not somatostatin- or calretinin-expressing, interneurons reduced theta power in the BLA model. These results suggest that long-range BF GABAergic projections may modulate network activity at their target regions through the formation of a common interneuron-type and oscillatory phase-specific disinhibitory motif.

## Introduction

Cholinergic, GABAergic, glutamatergic, and peptidergic neuronal groups in the basal forebrain (BF) innervate a wide array of cortical areas and subcortical limbic regions, including the hippocampal formation and the amygdaloid complex (Mesulam et al. 1983; Frotscher and Léránth 1985; Freund and Antal 1988; Zaborszky et al. 1999, 2015; Mascagni and McDonald 2009; Muller et al. 2011; McDonald et al. 2011; Agostinelli et al. 2019). Among these, the roles of medial septal cholinergic and GABAergic innervation of the hippocampus are well characterized in several cognitive functions and in generating hippocampal oscillatory rhythms (Frotscher and Léránth 1985; Freund and Antal 1988; Pang et al. 2001; Buzsáki 2002; Xu et al. 2004; Yoder and Pang 2005; McNaughton et al. 2006; Roland and Savage 2009; Hangya et al. 2009; Vega-Flores et al. 2014; Roland et al. 2014; Király et al. 2023). The BF also sends equally dense projections to the amygdaloid complex originating from the ventral pallidum (VP) and the substantia innominata (SI) (Carlsen et al. 1985; Mascagni and McDonald 2009; McDonald et al. 2011; Agostinelli et al. 2019; Fu et al. 2020). However, neither the mechanistic role of BF projections in causing amygdala oscillations nor its role in the affective processes orchestrated by the amygdaloid complex are well understood. Here, we adopted a two-pronged approach to characterize the anatomical structure and functional role of the BF innervation of the amygdaloid complex. We initially performed retrograde labeling and immunohistochemical characterization of BF neurons that project to various nuclei of the amygdala and to the bed nucleus of stria terminalis (BNST). Subsequently, we confirmed the VP/SI to BLA connectivity using viral anterograde tracing. Once we revealed the major source of cholinergic and non-cholinergic innervation of the amygdaloid complex, we used this information to develop a biophysically realistic amygdala network model to test the role of BF in the emergence of basolateral amygdala (BLA) network activity and theta oscillations in silico.

A subpopulation of GABAergic neurons located in the VP and SI target the basal (BL); and to a lesser degree, the lateral (LA), basomedial, and the central nuclei (CeA) of the amygdaloid complex, as well as the BNST (Carlsen et al. 1985; Mascagni and McDonald 2009; McDonald et al. 2011; Mongia et al. 2016; Agostinelli et al. 2019). The cholinergic to non-cholinergic ratio of the BLA-projecting BF neurons is estimated to be approximately 3:1 (Carlsen et al. 1985), with the GABAergic neurons making up at least 10% of the complete projections (Mascagni and McDonald 2009). The organization and function of non-cholinergic BF afferents in the limbic system have largely been explored within the context of the hippocampus. Septo-hippocampal GABAergic projections exclusively target GABAergic interneurons in the hippocampus (Freund and Antal 1988; Unal et al. 2015b), driving the local theta oscillations by forming interneuron type- and time/oscillatory phase-specific disinhibitory circuits with pyramidal neurons (Tóth et al. 1997; Yoder and Pang 2005; Hangya et al. 2009; Király et al. 2023). As with the septo-hippocampal GABAergic neurons, a large majority of VP/SI GABAergic amygdalopetal neurons form synapses selectively with GABAergic interneurons in the amygdala (McDonald et al. 2011). This suggests that BF GABAergic projections that target different limbic structures may possess shared structural features and circuit-level functions (Tóth et al. 1997; Unal et al. 2018). BF GABAergic innervation of the BLA may follow a synchronized rhythmic pattern (Hegedüs et al. 2021), as observed in the septo-hippocampal GABAergic neurons that exhibit individual theta-range rhythmicity (Varga et al. 2008; Hangya et al. 2009), as well as behavioral state dependent phase-coupling to the network oscillation in the target region (King et al. 1998; Joshi et al. 2017; Unal et al. 2018; Espinosa et al. 2019; Joshi and Somogyi 2020; Kocsis et al. 2022).

Based on the anatomical commonalities between the septo-hippocampal GABAergic projections and the amygdaloid complex-targeting VP/SI GABAergic neurons, we theorize that BF GABAergic neurons serve a common function in all downstream limbic structures via the formation of similar interneuron-type and oscillatory phase-specific disinhibitory circuits (Tóth et al. 1997; Yoder and Pang 2005; Hangya et al. 2009). As with septo-hippocampal GABAergic projections being integral to diverse hippocampal functions and to the underlying network activity, we postulate that the VP/SI GABAergic innervation of the BLA is essential for regulating amygdaloid computations (Sah and Westbrook 2008; Shin and Liberzon 2010) and associated theta oscillations implicated in fear learning (Pape and Driesang 1998; Pape et al. 1998; Seidenbecher et al. 2003; Lesting et al. 2011; Stujenske et al. 2014; Davis et al. 2017).

Here, we report findings from an anatomical-computational investigation of this theory. First, we have identified and quantified parvalbumin (PV)- or calbindin (CB)-immunoreactive (+) putative GABAergic or glutamatergic, and choline acetyltransferase (ChAT)-immunoreactive cholinergic BF neuronal subgroups that target the *input* (LA and BL) and *output* (CeA and BNST) centers of the amygdaloid complex. Based on these findings and previous anatomical data, we then developed a 1000-cell biophysical computational BLA network model that incorporated principal neurons and three different interneuron groups (Hummos and Nair 2017; Feng et al. 2019). The model featured rhythmic GABAergic and non-rhythmic cholinergic BF afferents, along with constant excitatory thalamic/cortical inputs. We used this model to investigate the contribution of BF projections to the generation of network oscillations within the BLA, a key nucleus situated at the core of the widely recognized *fear circuit* (Sah and Westbrook 2008; Shin and Liberzon 2010).

## Materials and methods

### Neuroanatomical experiments

#### Animals

Adult male Wistar rats (280–380 g; n = 15) were housed in standard cages with ad libitum access to food and water under controlled laboratory conditions (21 ± 1 °C; 40–60% humidity; 12:12 day/night cycle, lights on at 8:00 AM). All experimental procedures were approved by the Boğaziçi University Institutional Ethics Committee for the Use of Animals in Experiments (BÜHADYEK) and carried out by licensed personnel.

#### Stereotaxic surgery and retrograde tract-tracing

Animals were anesthetized with either intraperitoneal (IP) injections of ketamine (80 mg/kg)—xylazine (13.3 mg/kg) (retrograde tracing experiments) or isoflurane (4% for induction, 1–2% for maintenance). Following induction of anesthesia, a local anesthetic (Vemcaine, 10%) and a povidone-iodine solution were applied to the shaved forehead before placing the animal in the stereotaxic frame (Kopf Instruments, USA). A homeothermic heating pad was used to monitor and maintain the body temperature at 36 °C. Craniotomies were performed above the anterior–posterior (AP) and medial–lateral (ML) coordinates of the target nuclei.

**Fig. 1:**
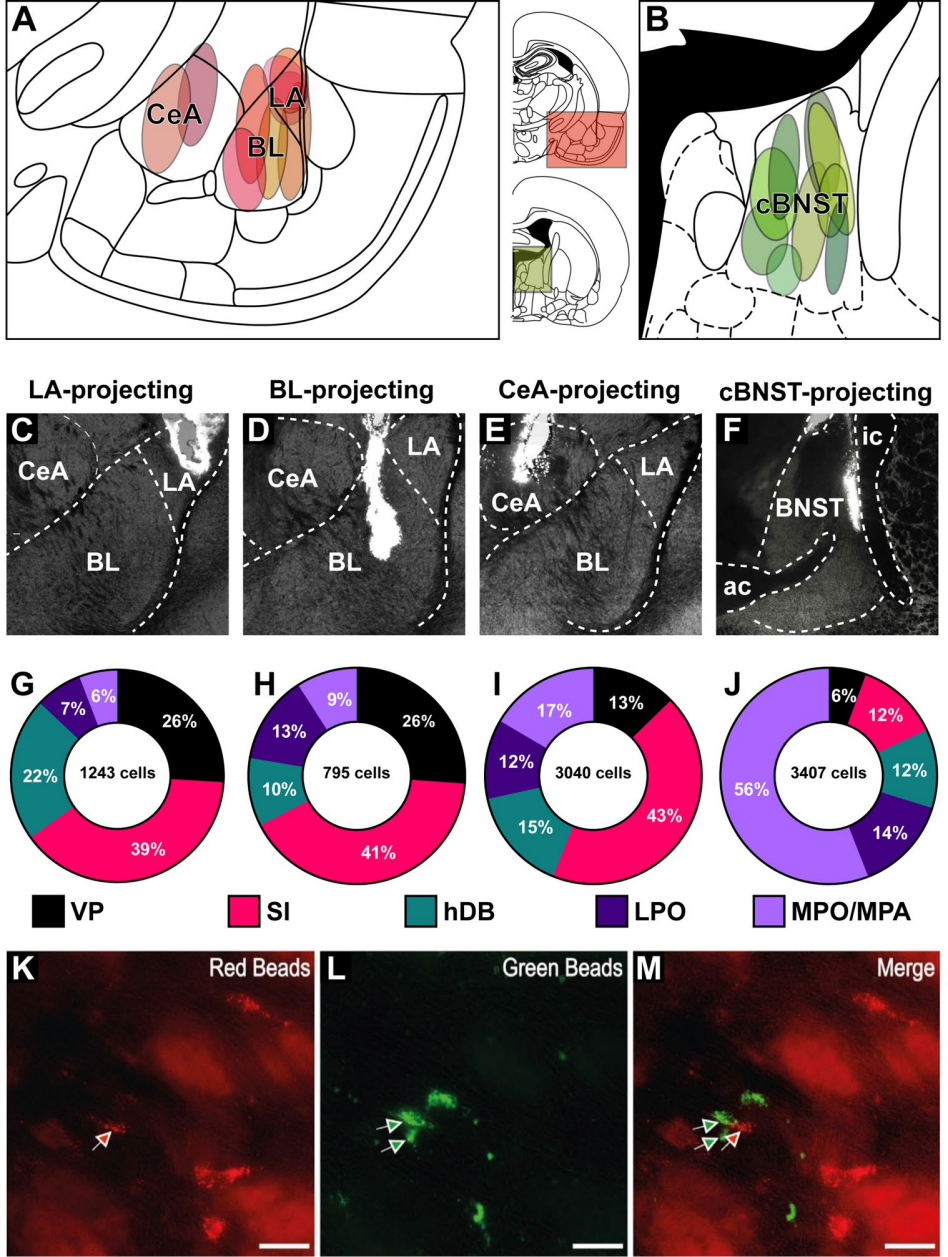
Retrobeads injections and resulting retrograde labeling in the basal forebrain and preoptic nuclei. **A**, **B** Injection sites targeting the amygdala nuclei (**A**) and the cBNST (**B**). **C**–**F** Brightfield photographs of representative injections in the LA (**C**), BL (**D**), CeA (**E**) and cBNST (**F**). **G**–**J** Donut charts demonstrating the percentage of LA (**G**), BL (**H**), CeA (**I**) and cBNST (**J**) targeting neurons in the observed basal forebrain and preoptic nuclei. **K**–**M** Fluorescent micrographs of retrogradely labeled neurons with red (**K**) or green (**L**) Retrobeads. *ac* anterior commissure, *BL* basolateral amygdala, *cBNST* central bed nucleus of stria terminalis, *CeA* central amygdala, *hDB* nucleus of horizontal limb of the diagonal band of Broca, *ic* internal capsule, *LA* lateral amygdala, *LPO* lateral preoptic area, *MPO/MPA* medial preoptic nucleus/area, *SI* substantia innominata, *VP* ventral pallidum

For retrograde tracing, red (diluted in saline by 1:2, volume = 200 nl) and green (undiluted, volume = 200 nl) fluorescent latex microspheres (Retrobeads, Lumafluor Inc., USA) were injected into the LA (AP = −2.80, ML = ± 5.30, DV = −7.30), BL (AP = −2.80, ML = ± 4.60, DV = −8.20), CeA (AP = −2.40, ML = ± 4.20, DV = −8.00), and the central BNST (cBNST) (AP = −0.48, ML = ± 1.40, DV = −6.00) (Paxinos and Watson 2007); Fig. 1A–B). In each animal, we injected one color of Retrobeads into one of the amygdaloid nuclei (LA, BL or CeA), while the other color of tracer was injected into the cBNST in the same hemisphere. Injections were performed unilaterally. The right and left target hemispheres were counterbalanced. The tracers (green or red Retrobeads) were also counterbalanced for each target region.

For anterograde tracing, AAV9-CaMKIIα-hM4D(Gi)-mCherry (250 nl, 1 × 10^13^ vg/ml, Addgene plasmid # 50,477, bilateral injection, n = 4) or AAV1-hSyn-EGFP-cre (250 nl, 1 × 10^13^ vg/ml, Addgene plasmid #105,540, unilateral injection, n = 2) were injected into the VP (AP = −0.20, ML = ± 2.5, DV = −7.80) or SI (AP = −1.00, ML = ± 2.50, DV = −8.10), (Paxinos and Watson 2007); Fig. 4A–B). AAV serotype 1 (AAV1) causes robust anterograde transsynaptic expression, enabling visualization of downstream synaptic targets of regionally specified starter cells (Zingg et al. 2022). Here, we injected AAV1-hSyn-EGFP-cre into the VP to transsynaptically label downstream neurons in the amygdala.

All injections were made with a microinjection syringe pump and a 1 μL micro-injection syringe (Hamilton, NV, USA). Each tracer injection took 5 min (40 nl/m for Retrobeads and 50 nl/m for viral tracing), after which the syringe was maintained at the target location for 10 min before retrieval to minimize dorsal diffusion. Once the incision was sutured, a local analgesic (Anestol pomade, 5% lidocaine and Jetokain, 5 mg/kg) was applied to the cranial surface before the animal was removed from the stereotaxic apparatus. The animals underwent a 5-day post-surgical recovery period in order to ensure maximal axonal transport of the Retrobeads or a 14-day post-surgical recovery period to allow optimal viral-driven expression of fluorescent proteins.

#### Perfusion-fixation and tissue preparation

Following the recovery period, animals were deeply anesthetized with the ketamine (80 mg/kg)—xylazine (13.3 mg/kg) mixture (IP), and perfused transcardially with 0.9% saline and 4% depolymerized paraformaldehyde (PFA) in 0.1 M PBS. Removed brains were post-fixed in the same fixative solution for 24–48 h at 4 °C. They were thoroughly rinsed following post-fixation and transferred to 0.1 M PB for slicing. Serial 60–80 µm thick coronal sections were obtained using a Leica VT 1000S vibratome (Leica Microsystems, Germany).

#### Immunohistochemistry

We conducted immunofluorescence labeling as described previously (Unal et al. 2015b; Akmese et al. 2023; Kingir et al. 2023). PBS with 0.3% Triton X-100 (PBS-TX) was used in all solutions and rinsing procedures to achieve tissue penetration. Coronal sections were rinsed 3 times (10 min each) in PBS-TX, followed by 1 h blocking at room temperature (RT) in 20% Normal Horse Serum (NHS) or Normal Goat Serum (NGS), depending on the secondary antibody. The sections were then incubated for 72 h at 4 °C in PBS-TX containing the primary antibodies and 1% NHS/NGS (refer to Table 1 for primary antibodies). Following 3 × 10 min of rinsing, the sections were incubated in the secondary antibody solution containing 1% NHS/NGS in PBS-TX for 4 h at RT. Sections were subsequently mounted and cover-slipped using methyl salicylate (Sigma-Aldrich, MO, USA) and examined using an epifluorescence (Olympus BX43) or confocal microscope (Leica SP8, Leica Microsystems).

**Table 1:** Primary antibodies

Molecule	Host species	Dilution	Source, catalogue #	Immunogen
CB	Mouse	1:1000	Swant, 300	Purified calbindin
CB	Rabbit	1:1000	Swant, CB38	Recombinant rat calbindin D-28 k
ChAT	Goat	1:500	Chemicon (Merck), ab144p	Human placental ChAT
Leu-enkephalin	Rabbit	1:1000	Abcam, ab22619	Synthetic peptide corresponding to Leu-enkephalin conjugated to keyhole limpet haemocyanin
PV	Rabbit	1:2000	Abcam, ab11427	Purified parvalbumin
PV	Rabbit	1:5000	Swant, PV27	Purified parvalbumin
SATB1	Goat	1:1000	Santa Cruz, sc-5989	N-terminus of human SATB1

Retrogradely labeled neurons were tested for different molecules listed in Table 1. Immunoreactivity for PV, CB, or AT-rich sequence-binding protein-1 (SATB1) was examined to identify non-cholinergic, putative GABAergic or glutamatergic neurons. ChAT immunoreactivity was assessed to identify cholinergic neurons, and Leu-enkephalin was used as a regional marker for VP (Fig. 1B). We used the following secondary antibodies: goat anti-rabbit Alexa Fluor 405 (1:250; A31556, Invitrogen), donkey anti-rabbit Alexa Fluor 488 (1:250; ab150073; Abcam), donkey anti-goat Cy3 (1:250; 705-165-147; Jackson ImmunoResearch Laboratories), donkey anti-goat DyLight650 (1:1000, ab96938, Abcam).

Sections from each brain were stained with DAPI or cresyl violet to facilitate histological identification of injection sites and cytological differentiation of the BF nuclei. DAPI staining was performed by incubating sections in the DAPI solution (1:2000, D3571, ThermoFisher) for 10 min. The sections were rinsed in PBS 3 times (10 min each) at RT. For cresyl violet staining, the sections were mounted on slides 3 days before the procedure and incubated for 1 min at 40 °C immediately before the staining. Slides were transferred through 100% ethanol (EtOH) (2 min), two sets of xylenes (2 min each), 100% EtOH (2 min), 70% EtOH (2 min), 20% EtOH (2 min), dH2O (1 min), cresyl violet solution (0.5 g cresyl violet acetate and 15 ml acetic acid in 500 ml dH2O, 15 min), differentiation solution containing 70% EtOH and 10% acetic acid (10 s), differentiation solution containing 100% EtOH and 10% acetic acid (10 s), 100% EtOH (5 min) and two set of xylenes (5 min each). Slides were then cover-slipped using Entellan new (Merck) mounting medium and examined under a light microscope.

#### Microscopy

The initial observations were conducted utilizing Olympus cellSens Imaging Software v2.2 on an epifluorescence microscope (Olympus BX43) equipped with a monochrome CCD camera (Olympus XM10). The images were obtained with 4x (Plan Apochromat, N.A. = 0.02, Nikon), 10x (Plan Fluor, N.A. = 0.30, Nikon), and 20x (Plan Fluor, N.A. = 0.50, Nikon) objective lenses. The 4 × objective lens was used for histological analysis, and 10 × and 20 × objective lenses were used to locate retrogradely labeled neurons in the target basal forebrain nuclei. Four fluorescent filter sets (for DAPI, Alexa Fluor 488, Cy3, and Cy5) were used for the detection of Alexa Fluor 405 fluorophores and DAPI, Alexa Fluor 488 fluorophores and green Retrobeads, Cy3 fluorophores and red Retrobeads, and DyLight 650 fluorophores respectively.

Following wide-field microscopic observations, multichannel fluorescence images were acquired with a Leica SP8 confocal microscope (Leica Microsystems, Wetzlar, Germany) using the LAS X software (Leica Microsystems) at a minimum pixel resolution of 1024 × 1024. The images were obtained with 20x (Plan Fluotar, N.A. = 0.4, dry, Leica Microsystems) or 40x (Plan Apochromat, N.A. = 1.10, water-immersion, Leica Microsystems) objective lenses. We employed four distinct lasers with wavelengths of 405, 488, 552, and 638 nm, along with PMT or HyD sensors, to visualize the fluorescence signal. The pinhole size was configured to 1 Airy unit. In the acquisition of z-stacks, the z-stack step size was set at half the optical section thickness. Post-acquisition brightness and contrast adjustments were performed uniformly on the whole image using the “FIJI” distribution of the ImageJ software (Schindelin et al. 2012). No non-linear or selective image adjustments were made on the acquired images.

#### Anatomical quantification

Retrogradely labeled neurons were manually counted in every other coronal section containing the labeled basal forebrain or preoptic nuclei. Observations and counting were made in the ventral pallidum, substantia innominata, horizontal diagonal band (hDB), lateral preoptic nucleus (LPO), and hypothalamic medial preoptic nucleus/area (MPO/MPA). Neuron quantification in the rostral-most parts of the extended amygdala was included in the SI. Cell counts were added together to obtain a total labeled neuron value for each region of interest. Normalized counts were derived by dividing the total number of labeled somata quantified in each nucleus by the number of observed sections. A similar quantification method was followed for the immunolabeled neurons. For each section, we counted all the cell bodies that were immunoreactive for a molecular marker in each region and noted the number of neurons that showed colocalization with Retrobeads. For each BF nucleus, we then calculated the percentage of neurons expressing each tested molecular marker among the observed retrogradely labeled neurons projecting to the LA, BL, CeA or the cBNST. Drawings depicting the distribution of labeled neurons in the BF were made with a camera lucida. All figures were created using Adobe Illustrator (v 25.0).

We analyzed the normalized fluorescence intensity of anterogradely labeled axonal fibers using the FIJI distribution of ImageJ software (Schindelin et al. 2012). Specifically, we measured the fluorescence intensity of VP/SI axonal fibers within the LA, BL, the lateral CeA (encompassing the capsular [CeAc] and lateral [CeAl] subdivisions), and the medial CeA (CeAm) in both hemispheres. Fluorescence intensity is reported in arbitrary units (AU). Data from both hemispheres were pooled for statistical analyses. To compare the normalized density of VP/SI axonal fibers across amygdala subnuclei, we performed a Student’s t-test.

The density of transsynaptically labeled neurons was quantified along the rostrocaudal axis in serial sections spaced 250 μm apart. Neuronal counts were conducted in the LA, BL, the lateral division of the CeA (CeAc and CeAl), and CeAm. Linear regression analysis was used to evaluate changes in neuronal density along the rostrocaudal axis.

### Model implementation

#### BLA network model

A 1000-neuron network model of the BLA was developed to investigate the potential role of the dense non-cholinergic, putative GABAergic projection, originating from the VP/SI and targeting the input nuclei of the basolateral amygdala, namely the LA and BL. The model incorporated our observations and previous reports on the synaptic parameters of the intrinsic (within BLA) and extrinsic (afferents to the BLA) connectivity. All parameters were derived from available rat or mouse amygdala data. The model was developed using the Allen Institute’s Brain Modeling Toolkit (BMTK) with the NEURON 7.7 simulator (Carnevale and Hines 2006), with a fixed time step of 0.1 ms. Verification of network connectivity parameters and plot generation were performed using the Python package BMTools. For the analysis of neuron power spectral density (PSD) and frequency, we computed spectrums utilizing the Welch Periodogram method (pwelch in MATLAB). Subsequent analyses and plot generation were performed using standard Python codes. The complete model is accessible for download on GitHub at https://github.com/tjbanks/AmygdalaTheta.

#### Single cell models

For computational modelling, we used the most parsimonious model that sufficiently explains the phenomena explored (Bassett et al. 2018). We modeled two types of principal neurons (PNs) and the three most populous groups of GABAergic interneuron that have been connected to local oscillations in the amygdala. The resulting network was able to reproduce relevant in vitro and in vivo properties of the BLA as described later.

The principal neurons (n = 800) were divided into two electrophysiological subtypes as Type A (adapting; PN_A_) (n = 569) and Type C (continuous; PN_C_) (n = 231). These model cells were adapted from our prior work (Feng et al. 2016, 2019). The three major groups of interneuron consist of (1) PV + Basket cells (n = 93), (2) calretinin (CR +) interneurons that often co-express vasoactive intestinal polypeptide (VIP) (Mascagni and McDonald 2003) and include interneuron-specific interneurons (Rhomberg et al. 2018) and small cholecystokinin (CCK)-expressing cells (n = 56), and (3) somatostatin (SOM)-expressing interneurons that include neurogliaform cells (NGFC; n = 51). The proportion of each neuronal group (56.9% PN_A_, 23.1% PN_C_, 9.3% PV + interneuron, 5.6% CR + interneuron, and 5.1% SOM + interneuron) was derived from previous reports (McDonald and Mascagni 2001; Mascagni and McDonald 2003; McDonald 2020). The parameters of single cell models are listed in Table 2.

**Table 2:** Parameters of single cell models

	PV + interneuron		Type A principal neuron			Type C principal neuron			SOM + interneuron			CR + interneuron		
V-rest (mv)	−70.0		−70.4			−70.3			−70.0			−60.1		
Input resistance (MΩ)	371		127			128			290			321		
Time Const. (ms)	20		32			32			19			20		

	Soma	Dend	Soma	Apical	Distal	Soma	Apical	Distal	Soma	Dend1	Dend2	Soma	Dend1	Dend2
Cm (µF/cm^2^)	1	1	2.4	2.4	2.4	2.4	2.4	2.4	1.3	1.3	1.3	1	1.3	1.3
Ra (Ωcm)	3375	150	150	150	150	150	150	150	150	150	150	150	150	150
Conductance (S/cm^2^)														
gNabar	0.035	0.01	0.015	0.015	0.015	0.045	0.015	0.015	–	–	–	–	–	–
gKdrbar	0.008	0.003	0.002	0.002	0.002	0.002	0.002	0.002	0.026	0.026	0.026	0.03	0.03	0.03
gLeak	1	1	2.50E−05	4.71E−05	4.71E−05	2.50E−05	4.71E−05	4.71E−05	6.70E−05	6.70E−05	6.70E−05	6.00E−05	6.00E−05	6.00E−05
gNapbar	–	–	0.00056	0.00045	0.00056	0.00055	0.00044	0.00055	0.0011	0.0011	0.0011	0.0014	0.0014	0.0014
gHdbar	–	–	1.50E-05	1.50E-05	1.50E-05	1.50E-05	1.50E-05	1.50E-05	–	–	–	–	–	–
gCabar	–	–	5.50E-04	5.50E-04	5.50E-04	5.50E-04	5.50E-04	5.50E-04	–	–	–	–	–	–
gMbar	–	–	0.00224	0.00179	0.00224	0.00224	0.00179	0.00224	0.0015	0.0015	0.0015	0.0015	0.0015	0.0015
gsAHPbar	–	–	0.05	–	–	0.002	–	–	–	–	–	0.0006	0.0006	0.0006
gKapbar	–	–	0.002	–	0.002	0.002	–	0.002	–	–	–	–	–	–
gNat	–	–	–	–	–	–	–	–	0.08	0.08	0.08	0.09	0.09	0.09
gCal	–	–	–	–	–	–	–	–	–	–	–	0.02	0.02	0.02

Principal neurons had three compartments: soma (diameter 24.75 µm, length 25 µm), a proximal dendrite (diameter 3 µm; length 270 µm), and an apical dendrite (diameter 5 µm; length 555 µm) to match passive properties. The specific membrane resistance, membrane capacity, and cytoplasmic (axial) resistivity values were as follows: *R*_m_ = 40 ± 5 kΩ-cm^2^, C_m_ = 2.4 µF/cm^2^, and R_a_ = 150 Ω-cm. The leakage reversal potential (*E*_L_) was set to −75 ± 4 mV. This configuration resulted in a resting membrane potential (V_rest_) of −66 ± 4 mV for both types A and C cells. The input resistance (*R*_IN_) was 140 ± 20 MΩ and 360 ± 20 MΩ, and time constant (*τ*_m_) was ~ 30 ms and ~ 20 ms, for Type-C and Type-A cells, respectively. These values fell within the reported ranges observed in physiological studies (Washburn and Moises 1992). The soma and dendrite compartments had the following currents: leak (*I*_L_), voltage-gated persistent muscarinic (*I*_M_), high-voltage activated Ca^2+^ (*I*_Ca_), spike-generating sodium (*I*_Na_), potassium delayed rectifier (*I*_DR_), A-type potassium (*I*_A_) (Li et al. 2009; Power et al. 2011), and hyperpolarization-activated nonspecific cation (*I*_h_) current. In addition, the soma exhibited a slow apamin-insensitive, voltage-independent afterhyperpolarization current (*I*_sAHP_) (Power et al. 2011; Alturki et al. 2016). The axonal compartments had the following currents: leak (*I*_L_*),* high-threshold sodium *(I*_Na1.2_*),* low-threshold sodium *(I*_Na1.6_*)*, and potassium delayed rectifier (*I*_DR_) (Hu et al. 2009). PNs exhibited adaptation characteristics, modulated by the magnitude of the Ca^2+^-dependent K^+^ current, set at either 50 mS/cm^2^ for Type A or 0.2 mS/cm^2^ for Type C (Kim et al. 2013). PN models were equipped with features for low- and high-threshold oscillations, designed to closely replicate physiological parameters (Pape et al. 1998; Li et al. 2009; Kim et al. 2013; Feng et al. 2016).

The PV + interneuron model contained two compartments: a soma-axon (diameter 15 µm; length 15 µm) and a dendrite (diameter 10 µm; length 150 µm). Each compartment contained a fast Na^+^ (*I*_Na_) and a delayed rectifier K^+^ (*I*_DR_) current.

The passive membrane properties of PV + interneurons were characterized by a specific membrane resistance (*R*_m_) of 20 ± 1 kΩ-cm^2^, a membrane capacity (*C*_m_) of 1 µF/cm^2^, and distinct cytoplasmic resistivities (*R*_a_) for the soma (3375 Ω-cm) and dendrite (150 Ω-cm). The resulting V_rest_ was −70 mV, input resistance (*R*_IN_) was 371 MΩ, and time constant (*τ*_m_) was 20 ms. The resulting current injection responses fell within the ranges reported in earlier reports (Faber et al. 2001; Sah et al. 2003; Rainnie et al. 2006).

The CR + and SOM + interneuron models contained three compartments: a soma-axon (diameter 10 µm; length 20 µm) and two dendrites (diameter 3 µm; length 250 µm). Each compartment contained persistent Na + (*I*_NaP_), potassium delayed rectifier current (*I*_KDR_), voltage-gated persistent muscarinic current (*I*_M_), and transient sodium channel (*I*_Nat_). In addition, the CR + interneuron contained l-calcium current, fast Na + (*I*_Na_), h channel (*I*_H_), and voltage-independent afterhyperpolarization current (*I*_sAHP_). The CR + interneuron model exhibited a membrane resistance of 80 ± 1 kΩ-cm^2^, membrane capacity of 1 µF/cm^2^, and a compartmental resistance was 150 Ω-cm. The resulting V_rest_ was −60 mV, input resistance (*R*_IN_) was 321 MΩ, and time constant (*τ*_m_) was 20 ms, as documented in earlier work (Kawaguchi and Kubota 1996; Porter et al. 1998; Caputi et al. 2009). The passive membrane properties of the SOM + interneurons were as follows: *R*m = 80 ± 1 kΩ-cm^2^, *C*_m_ = 1.3 µF/cm^2^ and *R*_a_ = 150 Ω-cm. The resulting V_rest_ was −70 mV, input resistance (*R*_IN_) was 290 MΩ, and time constant (*τ*_m_) was 19 ms. These values were within the ranges reported for SOM-containing interneurons (Karagiannis et al. 2009; Sosulina et al. 2010; Fanselow and Connors 2010).

#### Intrinsic and synaptic currents

The dynamics for each compartment (soma-axon or dendrite) followed the Hodgkin-Huxley formulation as previously described (Kim et al. 2013) in Eq. 1,

1 $$C_{m} dV_{s} /dt = - g_{L} \left( {V_{s} - E_{L} } \right) - g_{c} \left( {V_{s} - V_{d} } \right) - \sum I_{cur,s}^{int} \sum I_{cur,s}^{syn} + I_{inj} ,$$

where ${V}_{s}/{V}_{d}$ are the somatic/dendritic membrane potential (mV), ${I}_{cur,s}^{int}$ and ${I}_{cur,s}^{syn}$ are the intrinsic and synaptic currents in the soma, ${I}_{inj}$ is the electrode current applied to the soma, ${C}_{m}$ is the membrane capacitance, ${g}_{L}$ is the conductance of the leak channel, and ${g}_{c}$ is the coupling conductance between the soma and the dendrite (similar term added for other dendrites connected to the soma). The intrinsic current ${I}_{cur,s}^{int}$*,* was modeled as ${I}_{cur,s}^{int}={g}_{cur}{m}^{p}{h}^{q}({V}_{s}-{E}_{cur})$, where ${g}_{cur}$ is its maximal conductance, *m* its activation variable (with exponent *p*), *h* its inactivation variable (with exponent *q*), and ${E}_{cur}$ its reversal potential (a similar equation is used for the synaptic current ${I}_{cur,s}^{syn}$ but without *m* and *h*). The kinetic equation for each of the gating variables *x* (*m* or *h*) takes the form

2 $$\frac{dx}{{dt}} = \frac{{x_{\infty } \left( {V,\left[ {Ca^{2 + } } \right]_{i} } \right) - x}}{{\tau_{x} \left( {V,\left[ {Ca^{2 + } } \right]_{i} } \right)}},$$

where ${x}_{\infty }$ is the steady state gating voltage- and/or Ca^2+^- dependent gating variable and ${\tau }_{x}$ is the voltage**-** and/or Ca^2+^**-** dependent time constant. The equation for the dendrite follows the same format with ‘*s*’ and ‘*d*’ switching positions in Eq. 1.

Excitatory transmission was mediated by AMPA and NMDA receptors, while inhibitory transmission was modeled via GABA_A_ receptors. The corresponding ionic currents were modeled by dual exponential functions (Destexhe et al. 1994; Durstewitz et al. 2000), as shown in Eqns. 3–5,

3 $$\begin{gathered} I_{{AMPA}} = w \times G_{{AMPA}} \times \left( {V - E_{{AMPA}} } \right) \hfill \\ G_{{AMPA}} = g_{{AMPA,max}} \times F_{{AMPA}} \times s\left( V \right) \times r_{{AMPA}} \hfill \\ r_{{AMPA}} \begin{aligned} {\rm c}\kern-8pt/ \quad \end{aligned} = ~\alpha Tmax_{{AMPA}} \times ON_{{AMPA}} \times \left( {1 - r_{{AMPA}} {\text{~}}} \right) - \beta _{{AMPA}} \times r_{{AMPA}} , \hfill \\ \end{gathered}$$

4 $$\begin{gathered} I_{NMDA} = w \times G_{NMDA} \times \left( {V - E_{NMDA} } \right) \hfill \\ G_{NMDA} = g_{NMDA,max} \times F_{NMDA} \times s\left( V \right) \times r_{NMDA} \hfill \\ r_{NMDA} \begin{aligned} {\rm c}\kern-8pt/ \quad \end{aligned} = \alpha Tmax_{NMDA} \times ON_{NMDA} \times \left( {1 - r_{NMDA} { }} \right) - \beta_{NMDA} \times r_{NMDA} , \hfill \\ \end{gathered}$$

5 $$r_{GABAa} \begin{aligned} {\rm c}\kern-8pt/ \quad \end{aligned} = \alpha Tmax_{GABAa} \times ON_{GABAa} \times \left( {1 - r_{GABAa} { }} \right) - \beta_{GABAa} \times r_{GABAa} ,$$

where *V* is the membrane potential (mV) of the postsynaptic compartment (dendrite or soma), *I* is the current injected into the compartment (nA), *G* is the synaptic conductance (µS), $w$ is the synaptic weight (unitless), and *E* is the reversal potential of the synapse (mV). *g*_*x,max*_ is the maximal conductance (µS), *F* implements short-term plasticity, and *r*_*x*_ determines the synaptic current rise and decay time constants based on the terms *αTmax* and β (Destexhe et al. 1994). The voltage-dependent variable *s*(*V*) which implements the Mg^2+^ block was defined as *s*(*V*) = [1 + 0.33 exp(−0.06 V)]^−1^ (Zador et al. 1990). The terms *ON*_*NMDA*_ and *ON*_*AMPA*_ were set to 1 when the corresponding receptor was open, and 0 when it was closed. Reversal potential, rise/decay time constants, and conductance for the model were derived from previously published data (Thomson and Deuchars 1997; Galarreta and Hestrin 1997; Mahanty and Sah 1998; Porter et al. 1998; Weisskopf et al. 1999; Feng et al. 2019). Synaptic weights (*w*) for all connections followed a log-normal distribution with a cutoff set at three times the mean to avoid non-physiological values. The parameters for ionic currents are detailed in Table 3.

**Table 3:** Ionic current parameters

Current	Gating variable	α	β	${x}_{\infty }$	τ_x_ (ms)
_*INa1*_	*p* = *3*	$\frac{-0.4(V+30)}{\text{exp}[-(V+30)/7.2]-1}$	$\frac{0.124(V+30)}{\text{exp}[(V+30)/7.2]-1}$	$\frac{\alpha }{\alpha +\beta }$	$\frac{0.6156}{\alpha +\beta }$
	*q* = *1*	$\frac{-0.03(V+45)}{\text{exp}[-(V+45)/1.5]-1}$	$\frac{0.01(V+45)}{\text{exp}[(V+45)/1.5]-1}$	$\frac{1}{\text{exp}\left[(V+50)/4\right]+1}$	$\frac{0.6156}{\alpha +\beta }$
_*IKdr1*_	*p* = *1*	$\text{exp}[-0.1144(\text{V}+15)]$	$\text{exp}[-0.0801(\text{V}+15)]$	$\frac{1}{\text{exp}\left[(-V-15)/11\right]+1}$	$\frac{50*\beta }{1+\alpha }$
_*IH2*_	*q* = *1*	$\text{exp}[0.0832(\text{V}+75)]$	$\text{exp}[0.0333\left(V+75\right)]$	$\frac{1}{\text{exp}\left[(V+81)/8\right]+1}$	$\frac{\beta }{0.0081(1+\alpha )}$
_*IKM3*_	*p* = *2*	$\frac{0.016}{\text{exp}[-(V+52.7)/23] }$	$\frac{0.016}{\text{exp}[(V+52.7)/18.8]}$	$\frac{1}{\text{exp}\left[(-V-52.7)/10.3\right]+1}$	$\frac{1}{\alpha +\beta }$
_*ICa3*_	*p* = *2*	–	–	$\frac{1}{\text{exp}\left[(-V-30)/11\right]+1}$	$\frac{2.5}{\text{exp}\left[\frac{-\left(V+37.1\right)}{32.3}\right]+\text{exp}\left[\frac{\left(V+37.1\right)}{32.3}\right]}$
	*q* = *1*	–	–	$\frac{1}{\text{exp}\left[(V+12.6)/18.9\right]+1}$	420
_*INap4*_	*p* = *1*	–	–	$\frac{1}{\text{exp}\left[(-V-48)/5\right]+1}$	$2.5+14*\text{exp}\left[-\left|V+40\right|/10\right]$
_*IsAHP3*_	*p* = *1*	$\frac{0.0048}{\text{exp}[-5{\text{log}}_{10}({\left[Ca\right]}_{i2})-17.5]}$	$\frac{0.012}{\text{exp}[2{\text{log}}_{10}\left({\left[Ca\right]}_{i2}\right)+20]}$	$\frac{\alpha }{\alpha +\beta }$	48
_*INa1.25*_	*p* = *3*	$\frac{-0.182(V+30)}{\text{exp}[-(V+30)/7]-1}$	$\frac{0.124(V+30)}{\text{exp}[(V+30)/7]-1}$		$\frac{1}{\alpha +\beta }$
	*q* = *1*	$\frac{-0.024(V+50)}{\text{exp}[-(V+50)/5]-1}$	$\frac{0.0091(V+75)}{\text{exp}[(V+75)/5]-1}$	$\frac{1}{\text{exp}\left[(V+72)/6.2\right]+1}$	$\frac{1}{\alpha +\beta }$
_*INa1.65*_	*p* = *3*	$\frac{-0.182(V+43)}{\text{exp}[-(V+30)/6]-1}$	$\frac{0.124(V+43)}{\text{exp}[(V+30)/6]-1}$	$\frac{\alpha }{\alpha +\beta }$	$\frac{1}{\alpha +\beta }$
	*q* = *1*	$\frac{-0.024(V+50)}{\text{exp}[-(V+50)/5]-1}$	$\frac{0.0091(V+75)}{\text{exp}[(V+75)/5]-1}$	$\frac{1}{\text{exp}\left[(V+72)/6.2\right]+1}$	$\frac{1}{\alpha +\beta }$
_*INaT6*_	*p* = *3*	$\frac{Ra(V+15)}{1-\text{ exp}[-(V+15)/7.2]}$	$\frac{Rb(-V-15)}{1-\text{ exp}[-(-V-15)/7.2]}$	$\frac{\alpha }{\alpha +\beta }$	$\frac{1}{\alpha +\beta }$
	*q* = *1*	$\frac{Rd(V+30)}{1-\text{ exp}[-(V+30)/1.5]}$	$\frac{Rg(-V-30)}{1-\text{ exp}[-(-V-30)/1.5]}$	$1+ \frac{1}{\text{exp}\left[(V+35)/4\right]}$	$\frac{1}{\alpha +\beta }$
_*ICaL6*_	*p* = *2*	$\frac{15.69(-V+81.5)}{\text{exp}[-(V+81.5)/10]-1}$	$0.29*\text{ exp}[-\frac{V}{10.86}]$	$\frac{\alpha }{\alpha +\beta }$	$\frac{1}{\alpha + \beta }$

#### Intrinsic connectivity

The neuronal composition of the BLA network model comprised 56.9% PN_A_ (n = 569), 23.1% PN_C_ (n = 231), 9.3% PV + interneurons (n = 93), 5.6% CR + interneurons (n = 56) and 5.1% SOM + interneurons (n = 51). The PNs possess mutual connections with all interneuron groups. PV + interneurons target somata of the PNs, as well as the SOM + interneurons and other PV + cells, but not the CR + group. CR + interneurons form inhibitory synapses on all other neuron groups, similar to PNs. SOM + interneurons, in contrast, only target PNs and avoid the other groups (Fig. 5A). The probability of unidirectional or reciprocal synaptic connections between PNs and interneurons was set to 16%. Axonal conduction delay was distance-dependent using a conduction velocity of 500 μm/ms. Synaptic connectivity parameters are listed in Table 4.

**Table 4:** Synaptic connectivity parameters

	Overall connectivity	Unidirectional	Bidirectional
PN to PN	2%	1.96%	0.04%
PN to PV	26.82%	11.24%	15.58%
PN to SOM	31.19%	29.17%	2.01%
PN to CR	18.43%	16.41%	2.02%
PV to PN	52%	36.42%	15.58%
PV to PV	22.92%	17.41%	5.50%
PV to SOM	9.80%	9.80%	–
SOM to PN	6.57%	4.55%	2.01%
CR to PN	11.59%	9.57%	2.02%
CR to PV	29.70%	29.70%	–
CR to SOM	75.25%	75.25%	–

#### Extrinsic connectivity

The network model integrates thalamic/cortical glutamatergic afferents to the BLA, cholinergic innervation from the basal forebrain, and GABAergic afferents from the VP/SI. Additionally, uncorrelated stochastic background input is applied to all model cells.

##### Input 1: thalamic/cortical glutamatergic afferents

We modeled the thalamic and cortical glutamatergic afferents of the BLA as independent 2 Hz Poisson trains, which were delivered to the PNs, SOM + interneurons and CR + interneurons (Fig. 5A). Given the limited thalamic/cortical glutamatergic input received by PV + neurons (Smith et al. 2000) and the absence of any impact on model outcomes upon the removal of this connection, the schematic figures omit this ineffective connection.

##### Input 2: cholinergic innervation

The cholinergic innervation was simulated by changing the relevant synaptic conductance values, following prior work (Hummos et al. 2014). Three levels of cholinergic tone were modeled: low acetylcholine (ACh) (0), baseline ACh (1), and high ACh (2). For affected synapses, the synaptic current was multiplied by a factor as listed below, for both *i*_*ampa*_ and *i*_*GABA*_. For example, in the case of *i*_*ampa*_, we get Eq. 6 below (replace ampa with GABA for the inhibitory synapses),

6 $$i_{ampa} = i_{ampa} *\left( {1 + b_{ACh} *\left( {ACh - 1} \right)} \right).$$

Here, b_ACh_ and ACh together control the strength and sign for the various cholinergic conditions. For instance, an ACh value of 2 allows b_ACh_ to influence *i*_ampa_ positively, to make no change with ACh = 1, and to influence *i*_ampa_ negatively with ACh = −0.2. The specific b_ACh_ values (same for all ACh cases) and the corresponding synapses were as follows: 0.3 for all the background synapses (to PNs, and PV + , SOM + and CR + interneurons), 0.3 for PV + interneuron-PN and VP/SI-PN, 0.4 for SOM + interneuron-PN and CR + interneuron-PN, and 0.3 for VP/SI-PV + interneuron synapses.

##### Input 3: VP/SI GABAergic rhythmic innervation

The rhythmic GABAergic input from VP/SI was modeled by using previously described methods (Fink et al. 2015). The input was assigned a specific frequency, and each cell exhibited “jitter” in its response to the input, simulating intercellular variability. Jitter was Gaussian normal distributed (N) for each cell, with zero mean and SD ${\sigma }_{jitter}^{2}$. The time of the jth event of neuron i was given by:

7 $$t_{j}^{i} = jT + N\left( {O, \sigma_{jitter}^{2} } \right).$$

A total of 893 afferent cells were designed to individually exhibit independent 2 Hz Poisson activity. The afferents project onto 800 PN and 93 PV + interneurons with an average convergence of 1 and 10.1 cells, respectively (McDonald et al. 2011). Two states were considered for these afferents. In the baseline or non-modulated state, each afferent was independent at 2 Hz as above. For the theta-modulated state, the firing rate of the afferents were modulated with a sine wave:

8 $$r\left[ t \right] = A*(\sin \left( {2*\pi *f*t} \right) + \phi ) + off ,$$

where $A=\frac{off}{\frac{1}{d}-1}$, $f$ is the frequency, $t$ is a vector representing time, $\phi$ is the phase, $off$ is the offset firing rate of the spike train being modulated and $0<d<1$ is the depth of modulation which represents the amplitude of the sine wave relative to $off$. We used a depth of modulation 0.7. To generate the spike train, a random vector x[t] was generated with values uniformly distributed between 0 and 1. A spike was generated if $x\left[t\right]\le r\left[t\right]dt$ where dt in our case was 0.1.

For experiments with theta-modulated VP input, jitter was applied at 8 Hz. For all other experiments, no jitter was applied, and the VP input was 2 Hz Poisson.

##### Input 4: background input to all cells

In order to replicate the membrane potential fluctuations observed in vivo, we implemented a point conductance input directly onto the soma, simulating stochastic background synaptic activity through the Ornstein–Uhlenbeck process (Destexhe et al. 2001). Specifically, stochastic background input had two independent components, excitatory and inhibitory, for PN and PV + , SOM + , and CR + cell groups, as modeled previously by our group (Feng et al. 2019). Conductance values, mean (SD), for excitatory and inhibitory inputs for the cell groups were as follows (in mS): PN_A_: 0.0032 (0.003), 0.021 (0.008); PN_C_: 0.0032 (0.003), 0.021 (0.008); PV: 0.00121 (0.00012), 0.00573 (0.00264); SOM: 0.00121 (0.00012), 0.00573 (0.00264); CR: 0.0032 (0.003), 0.021 (0.008).

#### Conduction delays

Conduction delay D between two connected cells was calculated in a distance-dependent manner using Eq. 9:

9 $$D = \frac{{\sqrt {\left( {x_{1} - x_{2} } \right)^{2} + \left( {y_{1} - y_{2} } \right)^{2} + \left( {z_{1} - z_{2} } \right)^{2} } }}{{A_{V} }},$$

where $({x}_{1},{y}_{1},{z}_{1})$ and $({x}_{2},{y}_{2},{z}_{2})$ are the coordinates of the pre- and post- synaptic neurons, respectively. ${A}_{V}$ is the axonal conduction velocity (0.5 m/s).

#### Short-term plasticity

Model AMPA and GABA synapses exhibited short-term synaptic plasticity. As in our prior model (Feng et al. 2019) we used previous reports (Mahanty and Sah 1998; Ali and Thomson 1998; Silberberg et al. 2004; Silberberg and Markram 2007; Minneci et al. 2007; Woodruff and Sah 2007; Fanselow et al. 2008; Cauli et al. 2014; Riedemann 2019) to model short-term depression and facilitation in the synapses (see Table 5).

**Table 5:** Parameters of short-term plasticity

Connection	Type: depressing (D) or facilitating (F)	Parameter value D or F	tauF	D1/D2	Time constants tauD1/tauD2
PN to PN, PN to PV + , PV + to PN, PV + to PV + , SOM + to PV + , CR + to PN, SOM + to PN	Depressing	D = 0.7	20	0.95/0.9	40/70
PN to CR + , PN to SOM +	Facilitating	F = 1.5	150	1/1	40/70

Short-term plasticity was implemented by *F* (for facilitation) in Eqns. 3–5. It was calculated as follows: $\tau \_F*dF/dt=1-F$ and was constrained to be ≥ 1. A constant f was determined f (≥ 1) to represent the amount of facilitation per presynaptic action potential reported in experimental studies. F was then updated after each stimulus as F → F*f. Between stimuli, F recovered exponentially back towards 1. The same approach was used for synapses with depression, except the constant d (instead of f) had two factors d1 and d2 with d1 being fast and d2 being slow subtypes, and d = d_1*d_2. It was also constrained to be ≥ 1.

#### LFP calculation

The *extracellular mechanism* in NEURON was used to calculate the transmembrane currents of each compartment (Carnevale and Hines 2006; Parasuram et al. 2016). This was then used to calculate the extracellular potential ${\varnothing }_{EP}$ as follows:

10 $$\emptyset_{EP} = \frac{I}{4\pi \sigma \Delta s}{\text{log }}\left| {\frac{{\sqrt {h^{2} + r^{2} } - h}}{{\sqrt {l^{2} + r^{2} } - l}}} \right|,$$

where *I* denotes the transmembrane current from that compartment, *∆s* the length of the line compartment, *r* the radial distance from the line, *h* the longitudinal distance from the end of the line, and $l=\Delta s+h$ the distance from the start of the line (Parasuram et al. 2016). We chose conductivity $\sigma$ of the extracellular medium as 0.3 S/m (Goto et al. 2010; Einevoll et al. 2013). These extracellular potentials were then summed across compartments (Lindén et al. 2014) at 0.5 ms resolution, to obtain the LFP as ${\varnothing }_{LFPs}$, using Eq. 11,

11 $$\emptyset_{LFPs} = \mathop \sum \limits_{N = 1}^{N\_neurons} \mathop \sum \limits_{i = 1}^{n\_source} \frac{{I_{Ni} }}{{4\pi \sigma \Delta s_{{N_{i} }} }}{\text{log}}\left| {\frac{{\sqrt {h_{{N_{i} }}^{2} + r_{{N_{i} }}^{2} } - h_{{N_{i} }} }}{{\sqrt {l_{{N_{i} }}^{2} + r_{{N_{i} }}^{2} } - l_{{N_{i} }} }}} \right|,$$

where *N*_*i*_ denotes *i*^th^ compartment of *N*^th^ neuron in the network. We scaled the LFP using a factor of 1000 to match the in vivo data, following the procedure in our prior amygdala model (Feng et al. 2019). The structure of the network was unchanged for the different model runs, including the ablation experiments, and so the same scale factor was used across the cases to ensure valid comparison of LFP characteristics.

#### Entrainment to LFPs

We determined the theta phase preference, or entrainment, of individual neuronal groups by bandpass filtering the LFPs in the 4–12 Hz band. For this purpose, we used a 2 pole Butterworth filter implemented with the MATLAB function filtfilt. This function performs forward and backward filtering to minimize phase distortions. Subsequently, we computed the Hilbert transform of the filtered LFP signal to detect the phase and amplitude at each instant (Amir et al. 2018). This procedure was utilized for each spike of each neuron to calculate the neuronal group firing probability across the BLA theta oscillation.

#### Computational experiments

We initially performed six computational experiments, labeled as Cases 1–6, in order to characterize the roles of BLA afferents in creating and modulating the theta rhythm. Each simulation run lasted 15 s, of which only the last 10 s were retained to avoid transients in the initial part. Each case was run with 10 random seeds and the averaged results are reported as mean and SD.

Case 1 (Baseline VP/SI GABA + Baseline ACh) refers to the baseline BLA network state with 2 Hz Poisson input from the thalamic/cortical afferents, a 2 Hz non-modulated Poisson input from the VP/SI GABAergic neurons, and a baseline cholinergic tone (ACh = 1). Case 2 (Theta-modulated VP/SI GABA + Baseline ACh) is the same as Case 1, but included theta-modulated VP/SI GABAergic inputs. Case 3 (Theta-modulated VP/SI GABA + High ACh) is the same as Case 2, but with an increased cholinergic tone. Case 4 (Theta-modulated VP/SI GABA + Low ACh) is the same as Case 2, but with a decreased cholinergic tone. Case 5 (Baseline VP/SI GABA + High ACh) is the same as Case 1, but with an increased cholinergic tone. Case 6 (Baseline VP/SI GABA + Low ACh) is the same as Case 1, but with a decreased cholinergic tone.

In another set of experiments, we sequentially inactivated the individual BLA interneuron groups to investigate their relative contributions to the peak theta power under Case 3 (rhythmic VP/SI GABAergic and high level of cholinergic input). This was done by disconnecting the efferent connections of the inactivated neuron group, which elevated the firing rates of the PNs. Consequently, we decreased the background input to the PNs to restore their firing rate to baseline levels, ensuring a fair comparison between the experiments. For instance, when PV + interneurons are inactivated, the firing rates of thalamic/cortical projections to PNs are modulated to restore the average firing rates of PN_A_ and PN_C_ cell groups to baseline levels of 0.45 and 0.6 Hz, respectively.

## Results

### Neuroanatomical investigation

#### Basal forebrain innervation of the amygdala and the cBNST

Histological assessment revealed that the surgeries resulted in successful local injections into the target nuclei with negligible dorsal or medial/lateral diffusion of Retrobeads to the neighboring regions (Fig. 1A–F). Retrobeads were retrogradely transported into the cell bodies localized in different brain regions, following injections into the amygdaloid complex (Fig. 1A) and the cBNST (Fig. 1B). The target amygdaloid nuclei and the cBNST received projections of varying density from several basal forebrain and preoptic nuclei (Fig. 1G–J). We have examined each nucleus of the BF and neighboring preoptic regions for retrogradely labeled neurons, and consistently observed sufficient labeling in the VP, SI, hDB, LPO, and MPO/MPA, irrespective of the injection site. Overall, we quantified 8485 basal forebrain neurons projecting to the LA (1243 cells from n = 3 animals), BL (795 cells from n = 4 animals), CeA (3040 cells from n = 2 animals), and the cBNST (3407 cells from n = 9 animals).

The LA, BL, and CeA received the densest basal forebrain input from the SI (39% of labeled cells for the LA, 41% for the BL, and 43% for the CeA; Fig. 1G–I). Ventral pallidum constituted the second largest source of BF innervation for the amygdaloid nuclei (26% of labeled cells for both the LA and the BL, and 13% for the CeA, Fig. 1G–I). The LA-projecting neurons were predominantly located in the basal forebrain nuclei (87%) with sparse labeling in the LPO (7%) and MPO (6%). Basal forebrain innervation of the BL followed a similar pattern: relatively dense SI (41%) and VP (26%) projections were followed by axonal projections originating from the LPO (13%), hDB (10%) and MPO/MPA (9%). Neurons projecting to the CeA were primarily located in the SI (43%), while the CeA received projections of relatively similar density from the MPO/MPA (17%), hDB (15%), and VP (13%). The most concentrated projections directed towards the cBNST, in contrast to the amygdaloid nuclei, predominantly originated from the MPO/MPA (56% of labeled cells; Fig. 1J). This was followed by projections from the LPO (14%), hDB (12%) and SI (12%), with a comparatively lower density of connections from the VP (6%; Fig. 1J). Notably, the majority of the SI neurons targeting the cBNST were localized in the dorsal portions of SI.

We observed several instances of proximal labeling of red and green beads (Fig. 1K–M). However, only 2 out of 8485 neurons were co-labeled with both red and green tracers in their cell bodies in the hDB. This observation indicates that amygdala-targeting BF axons rarely bifurcate to form connections with the cBNST, corroborating findings from previous studies (Bienkowski and Rinaman 2013; Mongia et al. 2016). It is important to note that observations in this work were focused on the basal forebrain and surrounding preoptic nuclei, and there may be extra-BF neurons that project both to the cBNST and different amygdaloid nuclei.

#### Neurochemical characterization of the retrogradely labeled basal forebrain neuronal groups

Neurochemical profiles of the retrogradely labeled BF neurons were identified with immunohistochemistry for ChAT and biomarker molecules that are localized in subpopulations of non-cholinergic, putative GABAergic or glutamatergic neurons. We tested a total number of 2172 neurons that were labeled in the VP, SI, or hDB following Retrobeads injections into the LA (435 neurons from 2 animals), BL (226 neurons from 2 animals), CeA (915 neurons from 2 animals) and the cBNST (596 neurons from 6 animals) for PV (1023 neurons), CB (1058 neurons), ChAT (489 neurons) or SATB1 (91 cells) immunoreactivity (Table 6).

**Table 6:** Number and proportion of retrogradely labeled PV + , CB + and ChAT + neurons

Target	Location	Cells tested for PV			Cells tested for CB			Cells tested for ChAT		
		*N*	PV +	(%)	*N*	CB +	(%)	*N*	ChAT +	(%)
LA	VP	97	5	5.2	65	0	0.0	61	18	29.5
	SI	31	0	0.0	73	5	6.8	24	7	29.2
	hDB	89	0	0.0	80	1	1.3	61	8	13.1
BL	VP	49	2	4.1	47	10	21.3	42	20	47.6
	SI	40	3	7.5	46	3	6.5	29	13	44.8
	hDB	18	0	0.0	26	0	0.0	16	7	43.8
CeA	VP	118	0	0.0	90	7	7.8	59	9	15.3
	SI	304	3	1.0	270	20	7.4	70	11	15.7
	hDB	65	1	1.5	68	3	4.4	24	5	20.8
cBNST	VP	13	0	0.0	6	0	0.0	16	2	12.5
	SI	98	0	0.0	142	9	6,3	22	4	18.2
	hDB	101	0	0.0	145	1	0.7	65	13	20.0
Total		1023	14	1.4	1058	59	5.6	489	117	23.9

Our observations revealed that, overall, ChAT-immunopositive neurons constituted 23.9% of all amygdala- or cBNST-targeting basal forebrain neurons in the VP, SI and hDB. Specifically, 1.4% of the examined basal forebrain neurons projecting to the LA, BL, CeA, or cBNST showed immunoreactivity for PV, while 5.6% of all tested basal forebrain projection neurons displayed CB immunoreactivity (Table 6). None of the retrogradely labeled neurons showed immunoreactivity for SATB1. Furthermore, no instances of double labeling were observed for PV and ChAT or CB and ChAT in any of the double-labeled sections.

We established that a significant proportion of neurons projecting to amygdaloid nuclei or the cBNST were immunopositive for ChAT (Table 6), irrespective of their location or target region. The BL received the densest cholinergic projection with 46.9% of all BL-projecting VP, SI and hDB neurons showing immunoreactivity for ChAT (47.6% in the VP, 44.8% in the SI, 43.8% in the hDB). This was followed by LA- (overall 22.6%; 29.5% in the VP, 29.2% in the SI, 13.1% in the hDB), cBNST- (overall 18.4%; 12.5% in the VP, 18.2% in the SI, 20.0% in the hDB), and CeA-projecting (overall 16.3%; 15.3% in the VP, 15.7% in the SI, 20.8% in the hDB) ChAT + BF neuronal subpopulations.

**Fig. 2:**
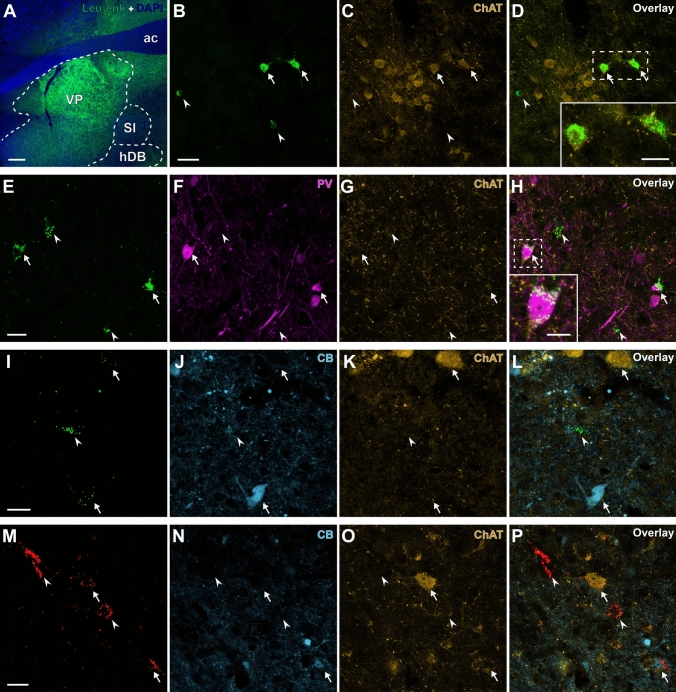
Confocal microscopic images of retrogradely labeled BF neurons (green or red) tested for ChAT (orange), PV (magenta) and CB (cyan) immunoreactivity. Arrows and arrowheads respectively point to retrogradely labeled neurons immunopositive or immunonegative for tested biomarker molecules. **A** Confocal microscopic tile-scan image showing dense labeling of Leu-enkephalin fibers in the VP. **B**–**D** LA-targeting neurons (**B**) tested for ChAT (**C**). Two retrogradely labeled ChAT + neurons (**D**) are enlarged in the inset. **E**–**H** BL-targeting neurons (**E**) tested for PV (**F**) and ChAT (**G**). One of the two labeled PV + neurons (**H**) is enlarged in the inset. **I**–**L** CeA-targeting neurons (**I**) tested for CB (**J**) and ChAT (**K**), showing one CB + and one ChAT + retrogradely labeled neuron. **M**–**P** cBNST-targeting neurons (**M**) tested for CB (**N**) and ChAT (**O**), showing one CB + and one ChAT + retrogradely labeled neuron. Scale bars: **A** 200 µm; **B**–**D** 40 µm; **D** inset, 20 µm; **E**–**H** 20 µm; **H** inset, 10 µm; **I**–**L** 15 µm; **M**–**P** 20 µm. *ac* anterior commissure, *hDB* nucleus of horizontal limb of the diagonal band of Broca, *SI* substantia innominata, *VP* ventral pallidum

**Fig. 3:**
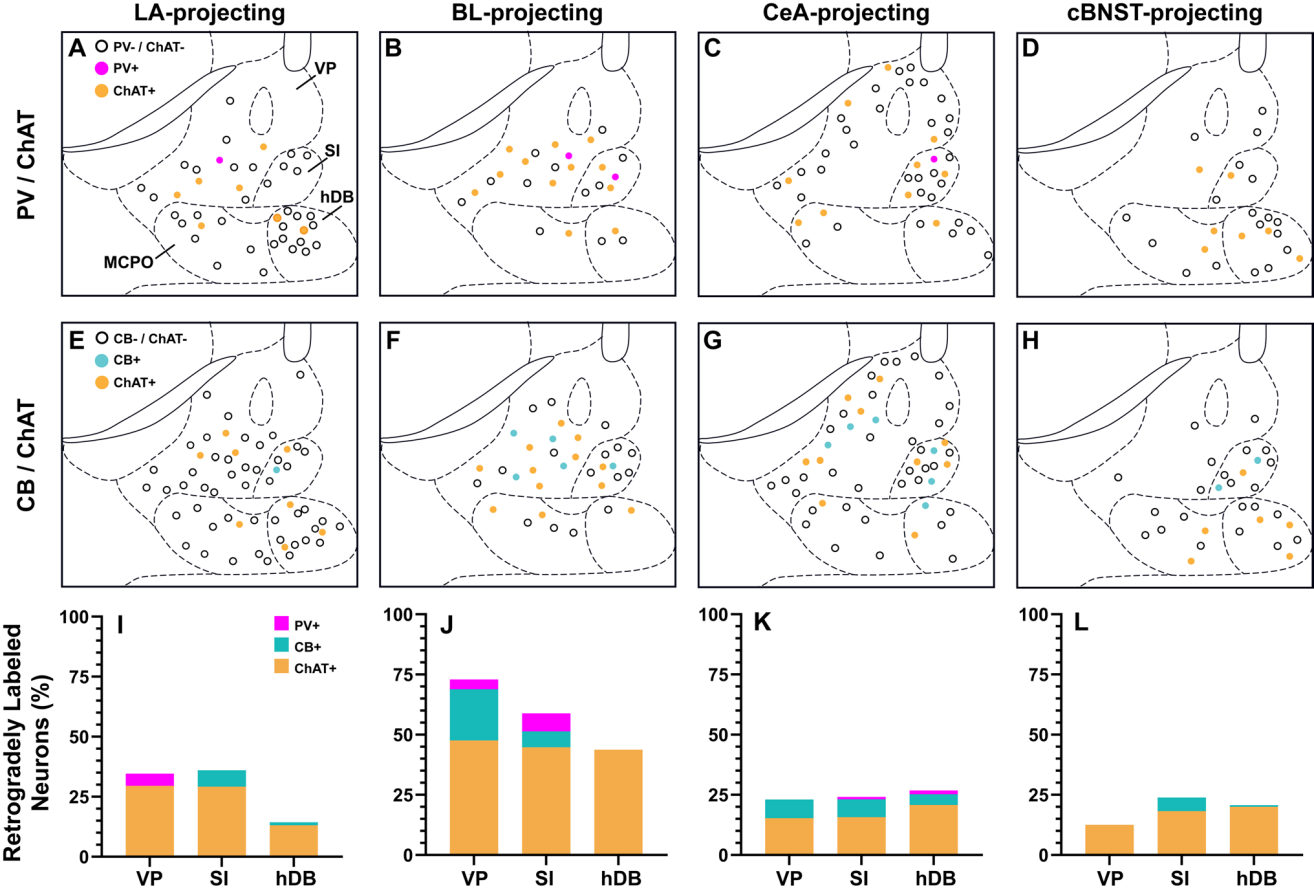
Distribution and proportion of retrogradely labeled neurons in the observed basal forebrain nuclei. **A**–**H** Schematic maps depicting LA (**A**, **E**), BL (**B**, **F**), CeA (**C**, **G**) and cBNST (**D**, **H**) targeting neurons tested for PV and ChAT (**A**–**D**) or CB and ChAT (**E**–**H**). Labeled cells in neighboring structures are omitted. **I**–**L** Percentage of PV (magenta), CB (cyan) and ChAT (orange) immunopositive cells within the tested LA (**I**), BL (**J**), CeA (**K**) and cBNST (**L**) targeting neurons

The immunohistochemical investigations (Fig. 2B–P, Table 6) further revealed that 5.25% of LA-projecting neurons and 4.1% of BL-projecting neurons in the VP were immunoreactive for PV. Interestingly, none of the tested LA-projecting SI neurons were PV-immunopositive. In contrast, 7.5% of the BL-targeting SI neurons were PV + (Figs. 2E–H, 3A–D, I–L). We did not observe any PV + LA- or BL-projecting neurons in the hDB. Within the CeA-projecting basal forebrain neurons, approximately 1% of labeled cells in the SI and 1.5% in the hDB were immunopositive for PV, while no PV + CeA-targeting neurons were observed in the VP. In contrast to the amygdala-targeting BF neurons, which involved a PV + subpopulation, no cBNST-projecting neuron in any of the observed nuclei (VP, SI, and hDB) showed immunoreactivity for PV.

We subsequently tested the retrogradely labeled basal forebrain neurons for CB immunoreactivity and observed retrogradely labeled CB-immunopositive cells in several nuclei (Fig. 3E–L, Table 6). Most significantly, CB + neurons constituted 21.2% of all projections to the BL originating from the VP and 6.5% of those originating from the SI. Similarly, 6.8% of LA-projecting SI neurons were immunopositive for CB. No labeled LA-projecting cell in the VP was CB-immunopositive. In the hDB, a minimal fraction of LA-projecting neurons displayed immunopositivity for CB (1.3%), and none among BL-projecting neurons was CB + . Small subsets of CeA-projecting neurons in all tested nuclei express CB (7.8% in the VP, 7.4% in the SI, 4.4% in the hDB). The cBNST-targeting CB + basal forebrain neurons were mostly localized to the SI with CB + neurons making up 6.3% of the cBNST-projecting-neurons in SI. Virtually no other cBNST-projecting BF neuron was immunopositive for CB (0.0% in the VP, 0.7% in the hDB).

A subpopulation of non-cholinergic SATB1-positive neurons has been identified in the hDB (Huang et al. 2011). We observed that a substantial portion (40%) of basal forebrain neurons targeting the cBNST were situated in the hDB. To investigate whether these neurons, identified as non-cholinergic SATB1 + neurons (Huang et al. 2011), project to the cBNST, we examined SATB1 immunoreactivity in cBNST-targeting basal forebrain neurons (n = 91). However, we did not observe any immunopositive neurons in the VP, SI, or the hDB.

These findings collectively unveil a dense non-cholinergic projection originating from the ventral pallidum and substantia innominata to the LA and BL nuclei of the amygdala. The neuronal population within the basal forebrain nuclei is known to encompass glutamatergic, GABAergic, and peptidergic neurons (Zaborszky et al. 1999), including neurons with the ability to co-express and corelease these neurotransmitters and acetylcholine (Nickerson Poulin et al. 2006; Saunders et al. 2015; Granger et al. 2016; Takács et al. 2018). Our results demonstrate that the neurochemical diversity observed in the basal forebrain extends to the substantial BLA-targeting projection of the VP/SI. Indeed, similar to the septo-hippocampal projections, a subset of these ChAT-negative neurons expressing PV is likely to be GABAergic. This suggests that the GABAergic VP/SI projection to the BLA may play a pivotal role in modulating local network activity, akin to the GABAergic septo-hippocampal innervation (Hangya et al. 2009; Király et al. 2023). In the subsequent computational investigation, we explored the potential role of this VP/SI GABAergic projection to the BLA using a biophysically realistic network model.

#### Anterograde tracing of VP/SI projections to the amygdala

**Fig. 4:**
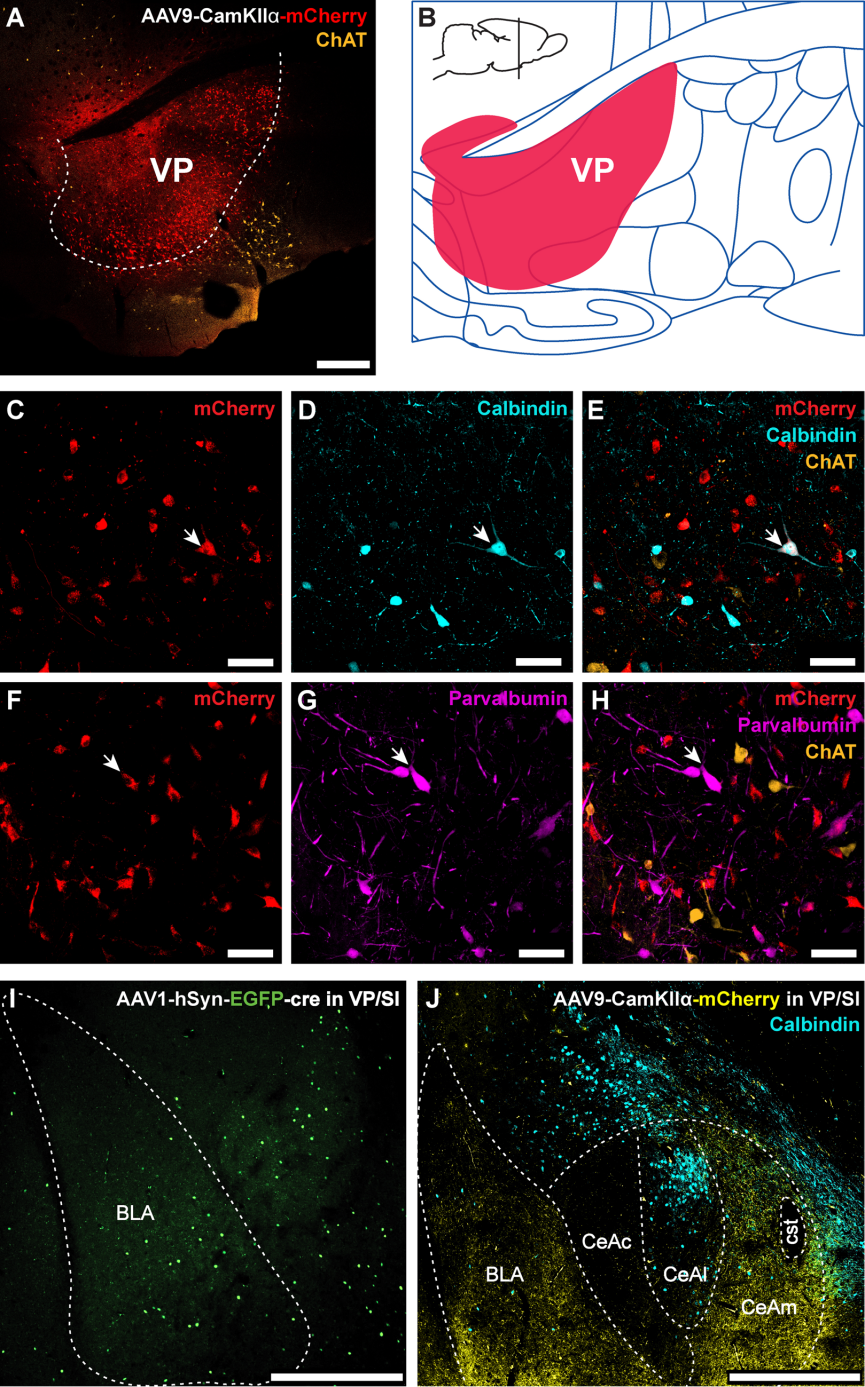
Anterograde and transsynaptic tracing of VP/SI projections to the amygdaloid complex. **A** AAV9-CaMKIIα-hM4D(Gi)-mCherry viral vector injection site in the ventral pallidum (VP). **B** Schematic showing spread of viral expression shown in panel (**A**). **C–H** VP mCherry-labeled neurons were tested for CB, PV, or ChAT immunoreactivity. Arrows indicate overlaps. **I** Transsynaptically labeled amygdala neurons following injection of AAV1-hsyn-EGFP-cre into VP/SI. **J** Innervation of the basolateral and central amygdala nuclei by mCherry + axonal fibers following injection of AAV9-CaMKIIα-hM4D(Gi)-mCherry into VP/SI. Calbindin immunolabeling delineates the borders of the capsular (CeAc), lateral (CeAl) and medial (CeAm) subdivisions of the central amygdala (cst: commissural stria terminalis). Scale bars: **A** 400 µm; **C**–**H** 50 µm; **I** 500 µm; **J** 500 µm

We conducted a series of anterograde and transsynaptic viral labeling experiments to ensure that the retrograde tracing results were not due to the retrograde tracer being taken up by passing fibers. Viral spread was histologically assessed in each experimental animal. In all cases, whether using AAV9-CaMKIIα-hM4D(Gi)-mCherry (n = 4) or AAV1-hSyn-EGFP-cre (n = 2) injections, viral expression was confined to both the VP (rostrolaterally) and the SI (caudomedially), with minimal spread into neighboring structures (Fig. 4A–B). The projection patterns observed were nearly identical across all animals, so the data for each viral vector were pooled as VP/SI injections before analysis.

We observed that CamKIIα promoter-driven mCherry expression was present in some, but not all, CB + , PV + , and ChAT + VP neurons (Fig. 4C–H). In all animals, dense axonal projections were observed in the BL and the medial subdivision of the CeA (CeAm), with no significant difference in fluorescence intensity between the BL (*M* = 82.81 ± 17.43 AU) and the CeAm (*M* = 70.80 ± 17.27 AU; *t*(14) = 1.384, *p* = 0.188). However, when compared to the BL, significantly less prominent yet consistent mCherry-labeled BF axons were detected in the LA (*M* = 29.41 ± 9.99 AU; *t*(14) = 7.518, *p* < 0.001; Fig. 4J). Interestingly, amygdala-projecting BF axons seemed to provide very sparse innervation to the capsular (CeAc) and the calbindin-rich lateral/intermediate (McDonald 1997) subdivision (CeAl), which showed significantly less axonal innervation (M = 16.71 ± 6.49 AU) compared to the CeAm (*t*(14) = 8.292, *p* < 0.001). In the BL, some mCherry-labeled VP/SI axonal boutons were observed in apposition to the somatic or dendritic compartments of CB + interneurons. These findings highlight the regional variability in VP/SI axonal projections within the amygdala.

Transsynaptic labeling using AAV1-hSyn-cre-EGFP (Zingg et al. 2022) further corroborated these findings. Consistent with the axonal intensity data, numerous EGFP + somata were detected in the BL (*M* = 131.87 ± 27.58 cells/mm^2^) and CeAm (*M* = 104.7263 ± 53.63 cells/mm^2^) following VP/SI injections (Fig. 4I). Fewer labeled neurons were observed in the ventromedial portion of the LA (*M* = *28.67* ± 18.53 cells/mm^2^) and the lateral division of the CeA (*M* = *27.61* ± 26.05 cells/mm^2^). Transsynaptically labeled neurons were distributed across the entire rostrocaudal extent of the BLA and CeA. To evaluate rostrocaudal distribution trends in each subnucleus, we performed linear regression analyses. Results showed that neuron density in the BL remained relatively stable along the rostrocaudal axis (*R*^*2*^ = 0.325, *F*(1, 6) = 2.883, p = 0.140). In contrast, significant decreases in neuronal density along the anterior-to-posterior axis were found in the LA (*R*^*2*^ = 0.567, *F*(1, 6) = 7.867, p = 0.031), CeAl (*R*^*2*^ = 0.742, *F*(1, 6) = 17.260, p = 0.006), and CeAm (*R*^*2*^ = 0.748, *F*(1, 6) = 17.780, p = 0.006). Furthermore, sparser transsynaptically labeled neurons were identified in the medial and basomedial amygdala nuclei as well as in the intra-amygdaloid division of the BNST.

### Computational investigation and predictions

#### Development and validation of the BLA network model

**Fig. 5:**
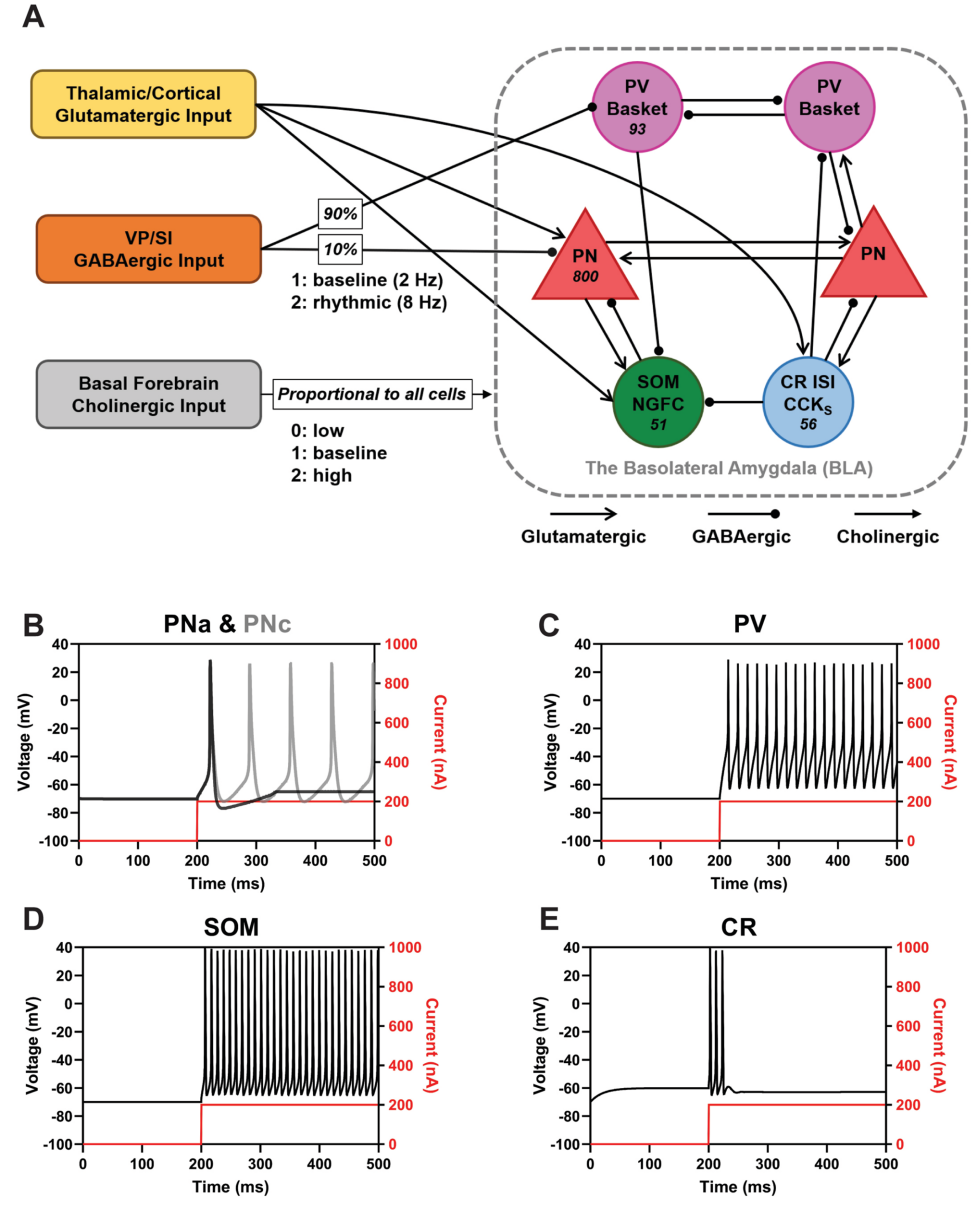
Extrinsic and intrinsic synaptic connectivity of the BLA network model, and single unit activity. **A** Schematic showing the synaptic connectivity of the BLA network model with principal neurons (PN) and the three most populous BLA interneuron groups: the parvalbumin (PV), somatostatin (SOM), and calretinin (CR) immunoreactive interneurons. **B** Action potential firing patterns of model amygdala neurons in response to continuous 200 nA current injections

We developed a biologically realistic, conductance-based network model of the amygdala that incorporated relevant cell types, as well as known ion channel and synaptic neurophysiology. Building on our previous model of the amygdala (Feng et al. 2016, 2019), we introduced single-cell models for two additional cell types—SOM + and CR + neurons—and connected them via synapses, with all properties derived from biological sources. The extrinsic inputs, intrinsic synaptic connectivity of the BLA network model, and sample current injection plots for different cell groups are shown in Fig. 5. Synapses between PNs and PV + interneurons, as well as between PNs and SOM + or CR + interneurons, exhibited connection-specific short-term synaptic plasticity (Table 5). The model was fine-tuned to replicate baseline firing rates reported in the literature (refer to Materials and Methods).

**Fig. 6:**
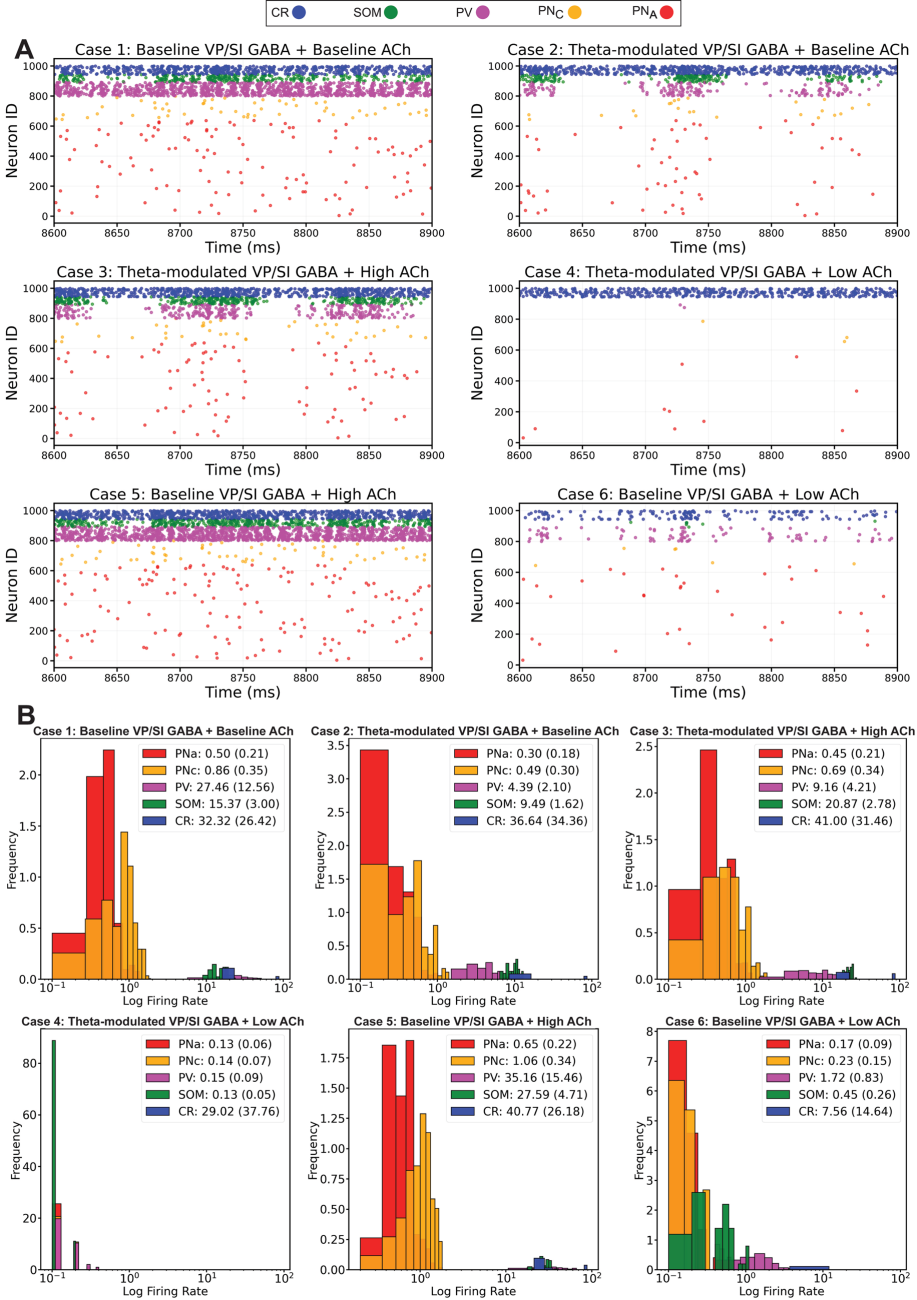
Single cell firing properties under different experimental cases. **A** Representative spike raster plots of model amygdala principal neuron and interneuron groups during a 300 ms period. **B** Histogram plots of the firing rates of different amygdala principal neuron and interneuron groups. Inset legends list firing rates (mean, SD, in Hz) of different neuronal groups

Finally, we confirmed that both the VP/SI GABAergic input rhythmicity (2 Hz vs. 8 Hz) and varying cholinergic tones (low, baseline or high level) produced the reported spread in single unit activity patterns of the BLA neuronal groups (Fig. 6).

#### VP/SI GABAergic and BF cholinergic projections differentially modulate BLA dynamics

After validating the BLA network model by assessing baseline unit activity, we explored how extrinsic inputs influenced network dynamics and theta oscillations. In all the model cases discussed below, the network configuration and parameters were consistently maintained, with the only adjustments being changes in synaptic weights between specific connections (refer to Materials and Methods).

In the baseline scenario (Case 1), characterized by a VP/SI input of 2 Hz random Poisson without modulation, no rhythmic firing was observed among the BLA cell groups (Fig. 6). Introducing modulation in Case 2 initiated rhythmicity, which further intensified in Case 3 with the High ACh state. However, theta power diminished with suppressed cholinergic tone in Case 4 (theta-modulated VP/SI GABA with Low ACh). BLA theta oscillation was entirely abolished in Case 5 (Baseline VP/SI GABA with High ACh) and Case 6 (Baseline VP/SI GABA with Low ACh), which incorporated baseline, non-rhythmic VP/SI inputs (Fig. 6).

**Fig. 7:**
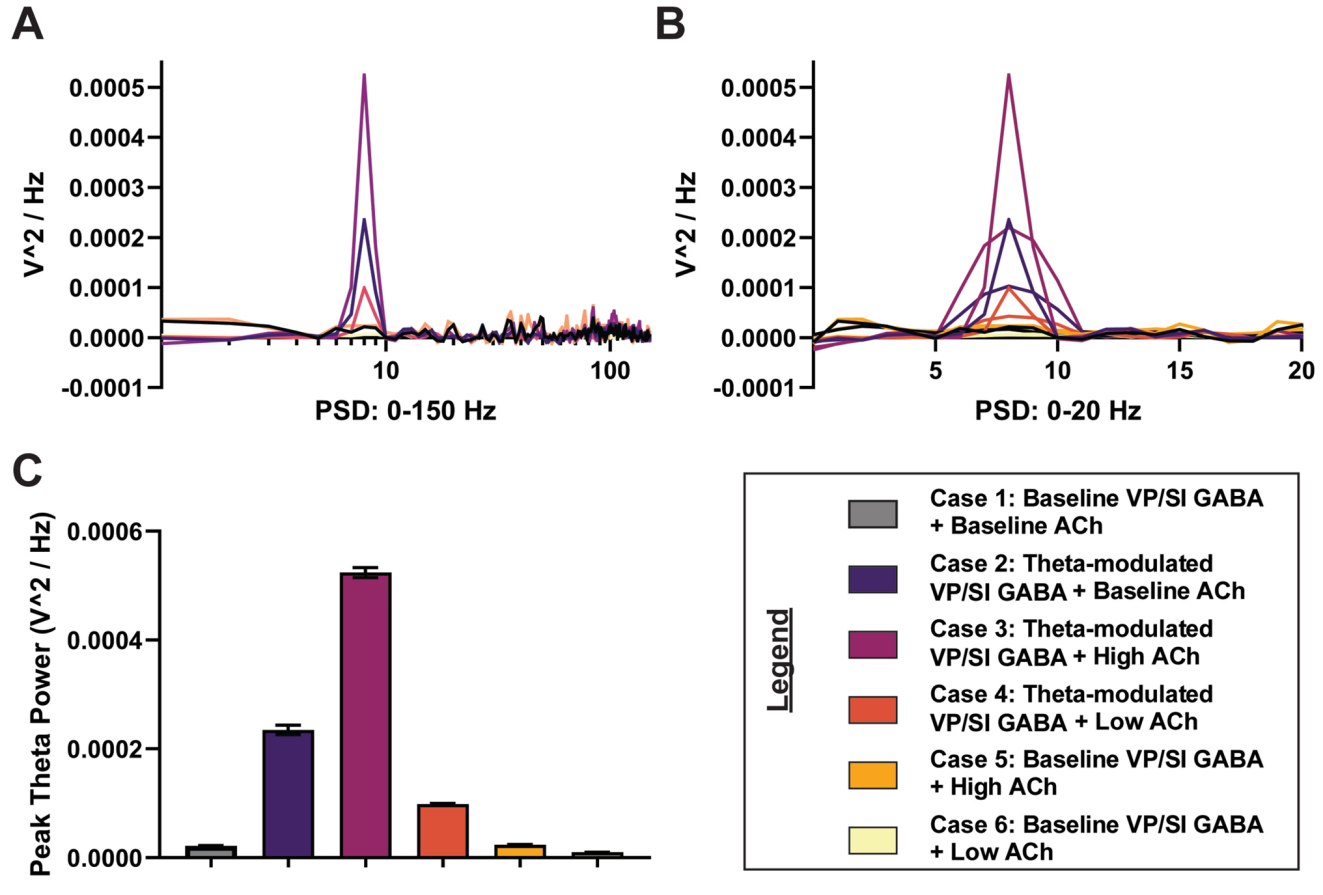
The PSD of the LFP for different experimental cases. **A** The PSD of the six experimental cases over 10 network instantiations across the 0–150 Hz band. **B** The PSD of the six experimental cases over 10 network instantiations across the 0–20 Hz band. **C** Peak theta power derived from the 0–20 Hz band. Error bars denote standard error of the mean

To further characterize the modulation of BLA theta, we computed the PSD of the LFP to which PNs serve as the primary contributors (Feng et al. 2019). The PSD plots for PNs reflect the power contributed to specific frequency bands by synchronous firing among these neurons (Fig. 7). As hypothesized, the absence of rhythmicity in VP/SI GABAergic inputs resulted in the absence of a detectable theta peak in the PSD for the baseline case. However, when rhythmicity was introduced in the VP/SI GABAergic input in Case 2, theta became evident in the PSD plot (Fig. 7). Increased cholinergic tone (Case 3) more than doubled the theta power, while low cholinergic innervation (Case 4) decreased it below baseline levels (Fig. 7). We further explored the impact of removing the rhythmic component of the VP/SI input at both High (Case 5) and Low (Case 6) ACh states. In both cases, we observed a considerable reduction in theta power, suggesting that cholinergic tone did not influence oscillatory power in the theta band of the model, in the absence of rhythmic VP/SI GABAergic input. Statistical results supported these observations, showing significant effects of both the ACh level (*F*(2, 54) = 925.1,* p* < 0.0001, 2 × 3 two-way ANOVA; Fig. 7C) and VP/SI GABAergic modulation (*F*(1, 54) = 3977,* p* < 0.0001) on the peak theta power, as well as a significant interaction between the effects of cholinergic and GABAergic inputs (*F*(2, 54) = 823.7,* p* < 0.0001).

**Fig. 8:**
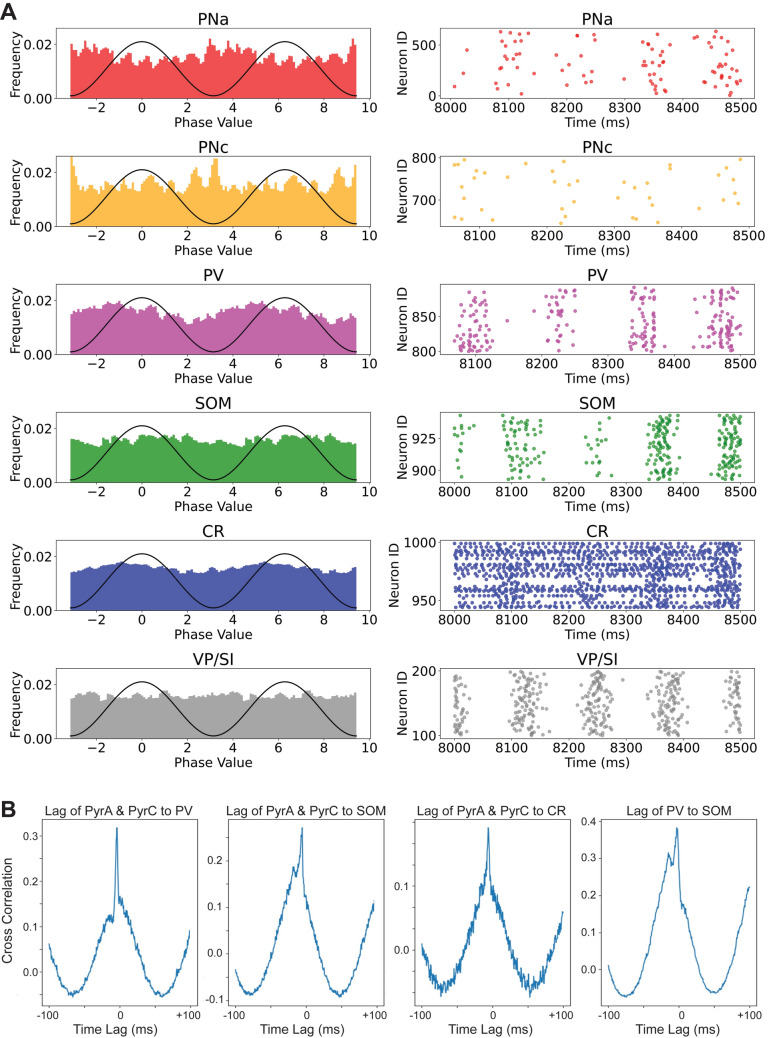
Theta phase-couplings and cross-correlograms of different principal neuron and interneuron groups for Case 2. **A** Spike histograms (left) and raster plots (right) depicting the theta-phase (sinusoidal wave) relationships of the modeled amygdala neuronal subpopulations and the VP/SI input. The frequency values were calculated using a rolling window size of 3 values over the phase values. **B** Coherence among amygdala neuronal groups

We subsequently investigated the entrainment of various cell groups to BLA theta using the Hilbert transform (see methods). As anticipated, both types of PNs exhibited higher spiking activity at the trough of the network theta when inhibition waned (Fig. 8A). The raster plots on the right side of Fig. 8A provide an estimate of the temporal spread of entrainment. PV + interneurons spiked about 45° after the trough, consistent with the majority of their drive originating from PNs. The SOM + interneurons followed them and increased their firing rate around the peak of the theta rhythm, while CR + interneurons were active in between PV + and SOM + cells (Fig. 8A).

We observed that the VP/SI input did not synchronize with network theta, as the LFP rhythm displayed fluctuating frequencies over time due to intrinsic interactions among various cell groups (Fig. 8A). An examination of theta cycles in the LFP unveiled substantial variations in both frequencies and amplitudes, as expected with intrinsic connectivity, demonstrating a frequency variation of 8 ± 4 Hz. Consequently, the VP/SI afferents, characterized by a narrow-band frequency of 8 ± 1 Hz, did not show any discernible phase preference. Cross-correlation estimates (Fig. 8B) revealed that PNs precede PV + , SOM + and CR + interneurons by 4.5, 6 and 6 ms, respectively. Also, PV + cells were observed to precede SOM + cells by 3 ms.

#### Ablation of BLA interneuron groups selectively alters network theta rhythm

**Fig. 9:**
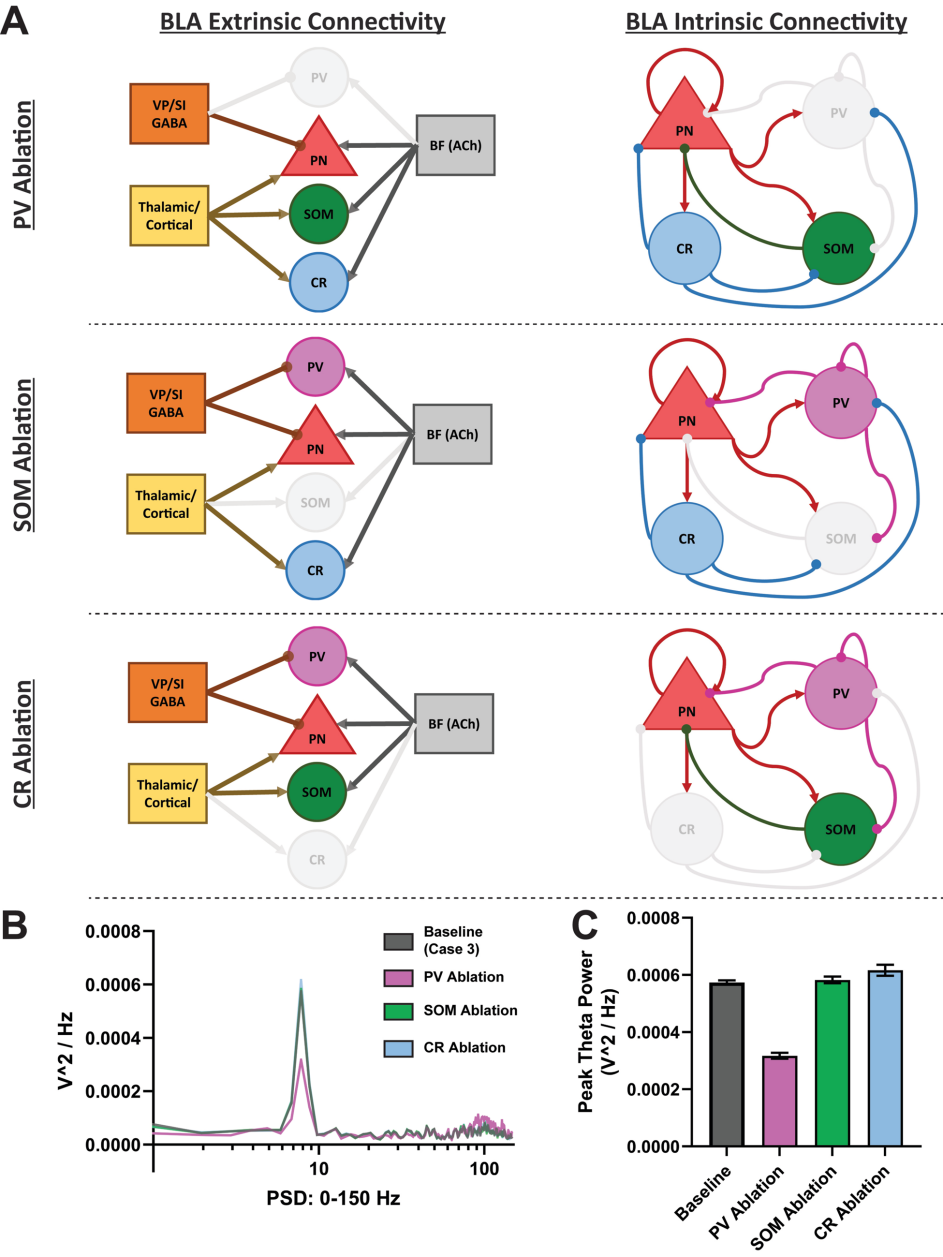
In silico ablation experiments conducted for Case 3 (theta-modulated VP/SI GABA + High ACh). **A** Schematic depiction of PV + (magenta), SOM + (green) and CR + (blue) neuronal group ablations. **B** The PSD of the four ablation cases over 10 network instantiations across the 40–120 Hz band. **C** Peak theta power at baseline (Case 3), and after PV + ablation, SOM + ablation, or CR + ablation. Error bars denote standard error of the mean

We explored the relative contributions of each interneuron group to BLA theta rhythm by individually inactivating them and documenting their effect on LFP theta under Case 3, where the strongest peak theta power was obtained (Fig. 9A). Results from model experimental runs were analyzed to calculate the firing rates of different neuron groups in the three ablation cases. We found a significant main effect of neuronal subtype ablations on the peak theta power in the BLA (*F*(3, 36) = 114.7,* p* < 0.0001, one-way ANOVA; Fig. 9C). Post-hoc comparisons showed that the PV + cells (*t*(36) = 14.07, *p* < 0.0001, Bonferroni corrected; Fig. 9C) emerged as the primary interneuron subtype responsible for LFP theta power in the BLA (Fig. 9B–C). This is likely attributable to the predominant convergence of afferent connectivity from VP/SI onto PV + interneurons (Fig. 5). PV + interneurons, in turn, inhibit BLA principal neurons, forming an oscillatory phase-specific disinhibitory circuit. Ablation of PV + interneurons disrupts this rhythmic disinhibition and reduces theta power in the BLA. In contrast, SOM + and CR + cells have minimal influence on LFP theta activity (Fig. 9B–C).

## Discussion

The present study investigated the source and target nuclei of the major non-cholinergic BF input to the amygdaloid complex, identifying substantial PV + or CB + projections from the VP/SI to the BLA. We subsequently tested the potential function of these non-cholinergic projections in a biophysically realistic BLA network model. Theta-modulation of VP/SI GABAergic inputs to the BLA facilitated local theta oscillations in the model, and the theta power was further modulated by increased cholinergic tone. Moreover, ablation of PV + interneurons in the BLA, while correcting for PN baseline firing rates, resulted in a significant reduction in theta rhythmicity within the BLA.

Network oscillations of the amygdala have been implicated in emotional arousal, fear learning, and affective memory processes (Paré and Collins 2000; Paré et al. 2002; Seidenbecher et al. 2003; Lesting et al. 2011; Stujenske et al. 2014; Davis et al. 2017). Several recent studies have established a connection between the synchrony of these oscillations across different brain regions and aversively motivated behavior (Karalis et al. 2016; Totty and Maren 2022; Totty et al. 2023). The septo-hippocampal GABAergic projection, the best-studied subcortical GABAergic projection in terms of its relationship to target-region LFP, modulates theta and supra-theta oscillations in the hippocampus (Hangya et al. 2009; Király et al. 2023). The network model findings in the present study indicate that another BF-limbic system connection, namely the VP/SI GABAergic projections to the BLA, may play a similar role in driving local network theta oscillations in the amygdala.

### Non-cholinergic innervation of the basolateral amygdala

The neuroanatomical experiments revealed that there are at least two different subpopulations of non-cholinergic projection neurons in the VP/SI that target different subnuclei of the amygdaloid complex. The PV + and CB + putative GABAergic or glutamatergic projection neurons collectively constituted around 25% of BL-projecting VP neurons and 14% of BL-projecting SI neurons. Moreover, our observations indicated a lower ratio of BL-targeting basal forebrain cholinergic neurons across various subnuclei compared to early reports, which suggested a ratio of up to 3:1 (Carlsen et al. 1985). In contrast to these earlier findings, another study utilizing Retrobeads in mice reported a cholinergic to non-cholinergic ratio among BL-projecting nucleus basalis of Meynert neurons closer to 64:36 (Aitta-aho et al. 2018). Specifically, in the VP and SI, we observed that approximately 45–48% of BL-targeting neurons were ChAT + , suggesting an approximate 1:1 ratio of cholinergic to non-cholinergic ratio amongst BL-targeting projection neurons in the adult male Wistar rat.

The proportions of PV + projection neurons reported in our study fall within the range of a previous report suggesting that approximately 4–13.1% of the VP/SI neurons targeting the BL are PV + (Mascagni and McDonald 2009). We found that approximately 4.1% of BL afferents from the VP, and 7.5% of those from the SI arise from PV + putative GABAergic or glutamatergic neurons. It should be noted that major subgroups of PV + VP projection neurons targeting the lateral habenula and the ventral tegmental area are glutamatergic (Knowland et al. 2017). Approximately 90% of PV + neurons in VP/SI are GAD + (Gritti et al. 2003), suggesting that some of the identified projection neurons are GABAergic. However, there is currently no conclusive evidence regarding the neurotransmitter profile of basal forebrain PV + neurons projecting to the BLA. The observation of PV + putative GABAergic VP/SI neurons densely projecting to the BLA, along with previous reports of selectively GABAergic BF-BLA projections (McDonald et al. 2011), provided a foundational basis for constructing a computational model of VP/SI-BLA innervation. It is also important to highlight that we did not observe a single instance of PV + projection neuron targeting the output centers of the amygdaloid complex studied here, namely the CeA or the cBNST.

In addition, we report a novel CB + VP neuronal population projecting primarily to the BL (21.3% of VP projections), and to a lesser extent, to the CeA (7.8%). These CB + neurons were immunonegative for ChAT, indicating a putative GABAergic or glutamatergic population of projection neurons. It is estimated that only 5% of CB + neurons are GAD-immunoreactive in the basal forebrain (Gritti et al. 2003), suggesting they are predominantly glutamatergic. However, cortically projecting CB + neurons exhibit earlier inhibitory responses compared to excitatory responses, implying they may provide long-range GABAergic projections that drive disinhibition of cortical neurons in the medial entorhinal cortex (Schlesiger et al. 2021). Thus, a subgroup of amygdala-projecting CB + neurons may be GABAergic. Additionally, it is possible that some of the identified CB + projection neurons are glutamatergic, resembling the cortical projection pattern observed in CB + BF neurons (Gritti et al. 2003). BF vGluT2 + neurons have also been reported to project to the amygdala (McKenna et al. 2021), but the neurochemical profiles of these neurons are unknown. Nevertheless, we showed that putative GABAergic or glutamatergic CB + neurons constitute a significant portion of the non-cholinergic VP-amygdala projections. Unlike the CB + VP projection neurons selectively targeting the BL and to a lesser extent the CeA, CB + projection neurons in the SI were found to uniformly innervate all observed nuclei of the amygdala. The CB + projections from the SI to the LA, BL, CeA, and cBNST exhibit a consistent CB + component, ranging from 5.7% to 7.4%. These neurons make up another non-cholinergic, putative GABAergic or glutamatergic (Gritti et al. 2003; McKenna et al. 2021), pathway from the BF to the amygdaloid complex.

Our observations of retrogradely labeled CeA-targeting BF neurons were made following injections that covered the medial (CeAm), lateral (CeAl), and capsular (CeAc) CeA subdivisions. Both Aitta-aho et al. (2018) and Barabás et al. (2024) show that the CeAm receives cholinergic input from the BF in mice. Our findings suggest that the retrogradely labeled cholinergic BF cells primarily project to the medial CeA, as our anterograde tracing reveals significantly fewer axons in the capsular or lateral subdivisions compared to the medial subdivision of the central amygdala. Barabás et al. (2024) also report LA-targeting retrogradely labeled BF cells in mice, though this population appears much smaller than BL-projecting BF neurons. Here, we show that a subpopulation of BF neurons project to the LA in rats and report that approximately one third of LA-projecting BF neurons are ChAT immunopositive. This result is line with numerous LA neurons being cholinoceptive in the rat brain (McDonald and Mascagni 2010, 2011). Thus, our retrograde tracing results suggest that part of the cholinergic innervation in the LA originates from the BF, while our anterograde tracing shows relatively sparse but consistent BF axonal projections to the lateral amygdala in rats. Small, targeted anterograde tracer injections in the rat basal forebrain could clarify the finer details of the topographical organization of the BF innervation of distinct nuclei within the amygdaloid complex.

Altogether, these results show that the VP/SI projections to the BLA originate from a diverse set of neuronal groups, comprising both cholinergic and non-cholinergic cells. The similarity between the BLA-projecting VP/SI neurons and the hippocampus-projecting neurons of the medial septum, in terms of their neurochemical profiles and synaptic selectivity, demonstrates a common circuit motif between the BF and target limbic structures.

### Common circuit motif in basal forebrain projections to limbic structures

The robust VP/SI projection to the BLA, along with its PV + and CB + subgroups, discovered in the present study aligns with the extensive variety of previously identified BF innervation observed in the amygdaloid complex (Carlsen et al. 1985; Mascagni and McDonald 2009; McDonald et al. 2011; Agostinelli et al. 2019; McKenna et al. 2021). However, the role of the non-cholinergic component of this versatile innervation on modulating network activity was largely unexplored. A recent computational model of the amygdala demonstrated that biophysically realistic models can generate network rhythmicity at the theta band with a minimal number of neurons (Cattani et al. 2023). Consequently, in our 1000-cell BLA model, we examined how the GABAergic VP/SI-BLA pathway, along with its interaction with cholinergic projections, modulates the local circuitry of the BLA, particularly concerning the generation of network theta oscillations. This enabled us to virtually test if the BF GABAergic innervation of the amygdala would have a function similar to that of the BF GABAergic innervation of another limbic structure, namely the GABAergic septo-hippocampal pathway.

The BLA-projecting GABAergic neurons of the VP/SI preferentially contact GABAergic interneurons (McDonald et al. 2011), akin to the pattern observed in GABAergic septo-hippocampal projections (Freund and Antal 1988; Unal et al. 2015b). The interneuron-preference observed in both the BF GABAergic projections implies a shared neuroanatomical organization and suggests a common mechanism involving the disinhibition of principal neurons in their respective target structures. Notably, septo-hippocampal connections are known to form oscillatory phase-specific disinhibitory circuits within their target hippocampal regions (Tóth et al. 1997; Yoder and Pang 2005; Hangya et al. 2009; Unal et al. 2018). Likewise, our computational model predicts that the interneuron-biased connectivity from VP/SI to BLA leads to the entrainment of BLA PV + neurons to the local theta rhythm (Fig. 7A). The PV + cells, in turn, entrain the PNs to the rhythm by providing them with windows of opportunity to fire, forming a disinhibitory circuit similar to the one orchestrated by the septo-hippocampal projections. Currently, there is no conclusive in vivo evidence that theta rhythms can be generated locally in the BLA. Thus, we do not rule out the possibility that BLA theta oscillations may at least partially originate from neighboring cortical structures and could be influenced by volume-conductance. BLA theta oscillation is not necessarily generated locally in vivo, but rather that the modelled BLA system entrains sufficiently to theta in the presence of theta-modulated BF GABAergic inputs. Therefore, our model indicates that if BLA can entrain to theta oscillations in vivo, the basal forebrain projection system—with its cholinergic and GABAergic components—may be well-suited to drive such oscillations, similar to the septo-hippocampal system.

The generation of a broad-band theta rhythm in the LFP was largely dependent on the interaction between PNs and PV + interneurons in our model. Notably, the excitatory inputs to PV + cells, primarily originating from PNs, play a significant role in modulating the theta rhythm, as illustrated in Fig. 8. At the circuit level, the broad-band theta rhythm results from several interconnected factors. An important consideration was the relative strength of the drive from the BF projections to BLA PV + neurons in comparison to the strengths of the drives among the intrinsic BLA connections, which add to the predictions of the model. For instance, we found that gamma generating mechanisms (PN-PV + -PN loop; Feng et al. (2019)) may need to be de-emphasized. Another was the interactions among interneuron subtypes, e.g., between SOM + and PV + groups, which had to be tuned (Feng et al. 2019). We note that the VP/SI afferents exhibit a narrow-band theta frequency at 8 Hz with jitter (ISI of 125 ± 25 ms). However, due to the local modulation of BLA theta by PN-PV + interneuron interactions, the VP/SI neurons lack a specific theta phase preference in LFP theta.

The phase preferences of various neuron groups within a network model of the hippocampus (Mysin et al. 2019) were shown to rely on specific afferents, and changes in relative phase relationships were sensitive to the number of afferents in the model. Thus, phase relationships can be modified by a single afferent, possibly originating from an unidentified source. In our amygdala network model (Fig. 5), we limit afferents to those from the thalamic/cortical and VP/SI regions, potentially neglecting other afferents (including neuromodulatory) that may weaken the gamma rhythm as indicated in the previous paragraph. This suggests that our computational model could serve as a testbed to investigate potentially unknown afferents during a particular brain state, if experimental data regarding the phase preferences of amygdala cells are available, akin to the approach employed in hippocampal studies (Somogyi et al. 2014). In the ablation experiments, we confirmed that the PV + cells, which selectively receive most of the rhythmic VP/SI input, were responsible for BLA theta power. In fact, the relative inhibitory power of PV + and SOM + interneurons may determine theta power in the amygdala (Bratsch-Prince et al. 2024).

### Functional significance of the basal forebrain GABAergic modulation

The basal forebrain cholinergic innervation of the amygdala has been studied extensively both in terms of its anatomical specifications and its behavioral functions (Woolf and Butcher 1982; Carlsen et al. 1985; Muller et al. 2011; Unal et al. 2015a; Gielow and Zaborszky 2017; Lee and Kim 2019; Kellis et al. 2020; Crimmins et al. 2022; Bratsch-Prince et al. 2024). As observed in the hippocampus (Lee et al. 1994; Vandecasteele et al. 2014), an increase in cholinergic tone in the BLA is associated with heightened local theta activity, both in vivo (Aitta-aho et al. 2018) and ex vivo (Bratsch-Prince et al. 2024). This suggests that BF neuronal function may be causally linked to the behavioral state-dependent theta oscillations in the amygdala. Likewise, the hippocampal theta oscillations are well-correlated with hippocampal functions like spatial navigation (Buzsáki 2005; Buzsáki and Moser 2013) and long-term memory processes (O’Keefe 1993; Buzsáki 2002). Local theta rhythm of the BLA, as well as its synchrony with the hippocampus and the prefrontal cortex, are associated with amygdala-dependent fear learning and extinction (Seidenbecher et al. 2003; Lesting et al. 2011; Stujenske et al. 2014; Davis et al. 2017).

As in the hippocampus (Wulff et al. 2009; Amilhon et al. 2015; Chung et al. 2020), deficits or functional alterations in amygdala PV + interneurons may underlie amygdala oscillatory states (Amaya et al. 2024) or dysfunctions in acquisition, extinction, or retrieval of fear memories (Lucas et al. 2016; Davis et al. 2017; Krabbe et al. 2018; Ozawa et al. 2020; Yau et al. 2021; Fu et al. 2022). Theta-rhythmic VP/SI GABAergic inputs to the amygdala may modulate network theta-dependent fear memory processes by recruiting local PV + interneurons. Hence, normal functioning of the PV + subpopulation may be required for supra- or sub-threshold theta oscillations in the BLA.

These observations suggest that BF GABAergic innervation of the hippocampus and the amygdala may contribute to memory processes led by these structures via similar mechanisms. As in the case of the septo-hippocampal innervation (Hangya et al. 2009; Király et al. 2023), the GABAergic VP/SI projection to the BLA may possess an overarching role in regulating local network dynamics. Our biophysical BLA network model tested this hypothesis in silico, providing predictions related to the role of basal forebrain GABAergic innervation in BLA network activity and theta oscillations. By incorporating and fine-tuning state-related parameters, the model has the potential to aid in the exploration of the role of BF projections to the amygdala in the formation and consolidation of emotional memories (Skirzewski et al. 2011; Davis et al. 2017; Ozawa et al. 2020), fear aversion (Stephenson-Jones 2019; Stephenson-Jones et al. 2020; Farrell et al. 2021), or depressive-like behaviors (Skirzewski et al. 2011; Akmese et al. 2023).

## Conclusion

Based on the structural commonalities of basal forebrain projections to the hippocampus and amygdala, as well as the predictions from the BLA network model, we posit that BF GABAergic projections serve a shared function across all limbic areas they target. Specifically, the GABAergic innervation from VP/SI to the BLA may be critical for generating local network oscillations, including theta and theta-modulated gamma rhythms, both of which are essential for amygdala-dependent learning and memory processes (Paré and Collins 2000; Paré et al. 2002; Seidenbecher et al. 2003; Lesting et al. 2011; Stujenske et al. 2014; Davis et al. 2017; Ozawa et al. 2020). These predictions await in vivo testing to enhance our understanding of the mechanisms underlying theta oscillations in the BLA and the role of this network oscillation in amygdala-dependent behavioral functions and dysfunctions.

## Data Availability

The BLA network model is available for download through GitHub at https://github.com/tjbanks/AmygdalaTheta. Other data will be made available on request.

## References

[CR1] Agostinelli LJ, Geerling JC, Scammell TE (2019) Basal forebrain subcortical projections. Brain Struct Funct 224:1097–1117. 10.1007/s00429-018-01820-6 30612231 10.1007/s00429-018-01820-6 PMC6500474

[CR2] Aitta-aho T, Hay YA, Phillips BU et al (2018) Basal forebrain and brainstem cholinergic neurons differentially impact amygdala circuits and learning-related behavior. Curr Biol 28:2557-2569.e4. 10.1016/j.cub.2018.06.064 30100338 10.1016/j.cub.2018.06.064

[CR3] Akmese C, Sevinc C, Halim S, Unal G (2023) Differential role of GABAergic and cholinergic ventral pallidal neurons in behavioral despair, conditioned fear memory and active coping. Prog Neuropsychopharmacol Biol Psychiatry 125:110760. 10.1016/j.pnpbp.2023.110760 37031946 10.1016/j.pnpbp.2023.110760

[CR4] Ali AB, Thomson AM (1998) Facilitating pyramid to horizontal oriens-alveus interneurone inputs: dual intracellular recordings in slices of rat hippocampus. J Physiol 507:185–199. 10.1111/j.1469-7793.1998.185bu.x 9490837 10.1111/j.1469-7793.1998.185bu.x PMC2230767

[CR5] Alturki A, Feng F, Nair A et al (2016) Distinct current modules shape cellular dynamics in model neurons. Neuroscience 334:309–331. 10.1016/J.NEUROSCIENCE.2016.08.016 27530698 10.1016/j.neuroscience.2016.08.016 PMC5086448

[CR6] Amaya KA, Teboul E, Weiss GL et al (2024) Basolateral amygdala parvalbumin interneurons coordinate oscillations to drive reward behaviors. Curr Biol 34:1561-1568.e4. 10.1016/j.cub.2024.02.041 38479389 10.1016/j.cub.2024.02.041 PMC11003843

[CR7] Amilhon B, Huh CYL, Manseau F et al (2015) Parvalbumin interneurons of hippocampus tune population activity at theta frequency. Neuron 86:1277–1289. 10.1016/j.neuron.2015.05.027 26050044 10.1016/j.neuron.2015.05.027

[CR8] Amir A, Headley DB, Lee SC et al (2018) Vigilance-associated gamma oscillations coordinate the ensemble activity of basolateral amygdala neurons. Neuron 97:656. 10.1016/J.NEURON.2017.12.035 29420934 10.1016/j.neuron.2017.12.035 PMC5809002

[CR9] Barabás B, Reéb Z, Papp OI, Hájos N (2024) Functionally linked amygdala and prefrontal cortical regions are innervated by both single and double projecting cholinergic neurons. Front Cell Neurosci. 10.3389/fncel.2024.1426153 39049824 10.3389/fncel.2024.1426153 PMC11266109

[CR10] Bassett DS, Zurn P, Gold JI (2018) On the nature and use of models in network neuroscience. Nat Rev Neurosci 19(9):566–578. 10.1038/s41583-018-0038-8 30002509 10.1038/s41583-018-0038-8 PMC6466618

[CR11] Bienkowski MS, Rinaman L (2013) Common and distinct neural inputs to the medial central nucleus of the amygdala and anterior ventrolateral bed nucleus of stria terminalis in rats. Brain Struct Funct 218:187–208. 10.1007/s00429-012-0393-6 22362201 10.1007/s00429-012-0393-6 PMC3662982

[CR12] Bratsch-Prince JX, Warren JW, Jones GC et al (2024) Acetylcholine engages distinct amygdala microcircuits to gate internal theta rhythm. J Neurosci 44:e1568232024. 10.1523/JNEUROSCI.1568-23.2024 38438258 10.1523/JNEUROSCI.1568-23.2024 PMC11055655

[CR13] Buzsáki G (2002) Theta oscillations in the hippocampus. Neuron 33:325–340. 10.1016/S0896-6273(02)00586-X 11832222 10.1016/s0896-6273(02)00586-x

[CR14] Buzsáki G (2005) Theta rhythm of navigation: link between path integration and landmark navigation, episodic and semantic memory. Hippocampus 15:827–840. 10.1002/hipo.20113 16149082 10.1002/hipo.20113

[CR15] Buzsáki G, Moser EI (2013) Memory, navigation and theta rhythm in the hippocampal-entorhinal system. Nat Neurosci 16:130–138. 10.1038/nn.3304 23354386 10.1038/nn.3304 PMC4079500

[CR16] Caputi A, Rozov A, Blatow M, Monyer H (2009) Two calretinin-positive GABAergic cell types in layer 2/3 of the mouse neocortex provide different forms of inhibition. Cereb Cortex 19:1345–1359. 10.1093/cercor/bhn175 18842664 10.1093/cercor/bhn175

[CR17] Carlsen J, Záborszky L, Heimer L (1985) Cholinergic projections from the basal forebrain to the basolateral amygdaloid complex: a combined retrograde fluorescent and immunohistochemical study. J Comp Neurol 234:155–167. 10.1002/cne.902340203 3886715 10.1002/cne.902340203

[CR18] Carnevale NT, Hines ML (2006) The NEURON book. Cambridge University Press. 10.1017/CBO9780511541612

[CR19] Cattani A, Arnold D, McCarthy M, Kopell N (2023) Basolateral amygdala oscillations enable fear learning in a biophysical model. Elife 12:RP89519. 10.7554/eLife.89519.1 10.7554/eLife.89519 PMC11594530 39590510

[CR20] Cauli B, Zhou X, Tricoire L et al (2014) Revisiting enigmatic cortical calretinin-expressing interneurons. Front Neuroanat 8:52. 10.3389/fnana.2014.00052 25009470 10.3389/fnana.2014.00052 PMC4067953

[CR21] Chung H, Park K, Jang HJ et al (2020) Dissociation of somatostatin and parvalbumin interneurons circuit dysfunctions underlying hippocampal theta and gamma oscillations impaired by amyloid β oligomers in vivo. Brain Struct Funct 225:935–954. 10.1007/s00429-020-02044-3 32107637 10.1007/s00429-020-02044-3 PMC7166204

[CR22] Crimmins BE, Lingawi NW, Chieng BC et al (2022) Basal forebrain cholinergic signaling in the basolateral amygdala promotes strength and durability of fear memories. Neuropsychopharmacology 2022:1–10. 10.1038/s41386-022-01427-w 10.1038/s41386-022-01427-w PMC9938249 36056107

[CR23] Davis P, Zaki Y, Maguire J, Reijmers LG (2017) Cellular and oscillatory substrates of fear extinction learning. Nat Neurosci 20:1624–1633. 10.1038/nn.4651 28967909 10.1038/nn.4651 PMC5940487

[CR24] Destexhe A, Mainen ZF, Sejnowski TJ (1994) Morphological and electrophysiological properties of principal neurons in the rat lateral amygdala in vitro. Neural Comput 6:14–18. 10.1162/NECO.1994.6.1.14

[CR25] Destexhe A, Rudolph M, Fellous J-M, Sejnowski TJ (2001) Fluctuating synaptic conductances recreate in vivo-like activity in neocortical neurons. Neuroscience 107:13–24. 10.1016/S0306-4522(01)00344-X 11744242 10.1016/s0306-4522(01)00344-x PMC3320220

[CR26] Durstewitz D, Seamans JK, Sejnowski TJ (2000) Dopamine-mediated stabilization of delay-period activity in a network model of prefrontal cortex. J Neurophysiol 83:1733–1750. 10.1152/JN.2000.83.3.1733 10712493 10.1152/jn.2000.83.3.1733

[CR27] Einevoll GT, Kayser C, Logothetis NK, Panzeri S (2013) Modelling and analysis of local field potentials for studying the function of cortical circuits. Nat Rev Neurosci 14(11):770–785. 10.1038/nrn3599 24135696 10.1038/nrn3599

[CR28] Espinosa N, Alonso A, Lara-Vasquez A, Fuentealba P (2019) Basal forebrain somatostatin cells differentially regulate local gamma oscillations and functionally segregate motor and cognitive circuits. Sci Rep. 10.1038/S41598-019-39203-4 30796293 10.1038/s41598-019-39203-4 PMC6384953

[CR29] Faber ESL, Callister RJ, Sah P (2001) Morphological and electrophysiological properties of principal neurons in the rat lateral amygdala in vitro. J Neurophysiol 85:714–723. 10.1152/jn.2001.85.2.714 11160506 10.1152/jn.2001.85.2.714

[CR30] Fanselow EE, Connors BW (2010) The roles of somatostatin-expressing (GIN) and fast-spiking inhibitory interneurons in up-down states of mouse neocortex. J Neurophysiol 104:596–606. 10.1152/jn.00206.2010 20538767 10.1152/jn.00206.2010 PMC2934925

[CR31] Fanselow EE, Richardson KA, Connors BW (2008) Selective, state-dependent activation of somatostatin-expressing inhibitory interneurons in mouse neocortex. J Neurophysiol 100:2640–2652. 10.1152/jn.90691.2008 18799598 10.1152/jn.90691.2008 PMC2585405

[CR32] Farrell MR, Esteban JSD, Faget L et al (2021) Ventral pallidum GABA neurons mediate motivation underlying risky choice. J Neurosci 41:4500. 10.1523/JNEUROSCI.2039-20.2021 33837052 10.1523/JNEUROSCI.2039-20.2021 PMC8152612

[CR33] Feng F, Samarth P, Paré D, Nair SS (2016) Mechanisms underlying the formation of the amygdalar fear memory trace: a computational perspective. Neuroscience 322:370. 10.1016/J.NEUROSCIENCE.2016.02.059 26944604 10.1016/j.neuroscience.2016.02.059 PMC4805477

[CR34] Feng F, Headley DB, Amir A et al (2019) Gamma oscillations in the basolateral amygdala: biophysical mechanisms and computational consequences. eNeuro. 10.1523/ENEURO.0388-18.2018 30805556 10.1523/ENEURO.0388-18.2018 PMC6361623

[CR35] Fink CG, Gliske S, Catoni N, Stacey WC (2015) Network mechanisms generating abnormal and normal hippocampal high-frequency oscillations: a computational analysis. eNeuro. 10.1523/ENEURO.0024-15.2015 26146658 10.1523/ENEURO.0024-15.2015 PMC4487885

[CR36] Freund TF, Antal M (1988) GABA-containing neurons in the septum control inhibitory interneurons in the hippocampus. Nature 336:170–173. 10.1038/336170a0 3185735 10.1038/336170a0

[CR37] Frotscher M, Léránth C (1985) Cholinergic innervation of the rat hippocampus as revealed by choline acetyltransferase immunocytochemistry: a combined light and electron microscopic study. J Comp Neurol 239:237–246. 10.1002/cne.902390210 4044938 10.1002/cne.902390210

[CR38] Fu J-Y, Yu X-D, Zhu Y et al (2020) Whole-brain map of long-range monosynaptic inputs to different cell types in the amygdala of the mouse. Neurosci Bull 36:1381–1394. 10.1007/s12264-020-00545-z 32691225 10.1007/s12264-020-00545-z PMC7674542

[CR39] Fu X, Teboul E, Weiss GL et al (2022) Gq neuromodulation of BLA parvalbumin interneurons induces burst firing and mediates fear-associated network and behavioral state transition in mice. Nat Commun 13:1290. 10.1038/s41467-022-28928-y 35277502 10.1038/s41467-022-28928-y PMC8917207

[CR40] Galarreta M, Hestrin S (1997) Properties of GABAA receptors underlying inhibitory synaptic currents in neocortical pyramidal neurons. J Neurosci 17:7220. 10.1523/JNEUROSCI.17-19-07220.1997 9295368 10.1523/JNEUROSCI.17-19-07220.1997 PMC6573445

[CR41] Gielow MR, Zaborszky L (2017) The input-output relationship of the cholinergic basal forebrain. Cell Rep 18:1817–1830. 10.1016/j.celrep.2017.01.060 28199851 10.1016/j.celrep.2017.01.060 PMC5725195

[CR42] Goto T, Hatanaka R, Ogawa T et al (2010) An evaluation of the conductivity profile in the somatosensory barrel cortex of wistar rats. J Neurophysiol 104:3388–3412. 10.1152/JN.00122.2010 20810682 10.1152/jn.00122.2010

[CR43] Granger AJ, Mulder N, Saunders A, Sabatini BL (2016) Cotransmission of acetylcholine and GABA. Neuropharmacology 100:40–46. 10.1016/j.neuropharm.2015.07.031 26220313 10.1016/j.neuropharm.2015.07.031 PMC4584188

[CR44] Gritti I, Manns ID, Mainville L, Jones BE (2003) Parvalbumin, calbindin, or calretinin in cortically projecting and GABAergic, cholinergic, or glutamatergic basal forebrain neurons of the rat. J Comp Neurol 458:11–31. 10.1002/cne.10505 12577320 10.1002/cne.10505

[CR45] Hangya B, Borhegyi Z, Szilágyi N et al (2009) GABAergic neurons of the medial septum lead the hippocampal network during theta activity. J Neurosci 29:8094. 10.1523/JNEUROSCI.5665-08.2009 19553449 10.1523/JNEUROSCI.5665-08.2009 PMC6666051

[CR46] Hegedüs P, Heckenast J, Hangya B (2021) Differential recruitment of ventral pallidal e-types by behaviorally salient stimuli during Pavlovian conditioning. iScience 24:102377. 10.1016/j.isci.2021.102377 33912818 10.1016/j.isci.2021.102377 PMC8066429

[CR47] Hu W, Tian C, Li T et al (2009) Distinct contributions of Nav1.6 and Nav1.2 in action potential initiation and backpropagation. Nat Neurosci 12(8):996–1002. 10.1038/nn.2359 19633666 10.1038/nn.2359

[CR48] Huang Y, Zhang L, Song NN et al (2011) Distribution of Satb1 in the central nervous system of adult mice. Neurosci Res 71:12–21. 10.1016/J.NEURES.2011.05.015 21658419 10.1016/j.neures.2011.05.015

[CR49] Hummos A, Nair SS (2017) An integrative model of the intrinsic hippocampal theta rhythm. PLoS ONE 12:e0182648. 10.1371/JOURNAL.PONE.0182648 28787026 10.1371/journal.pone.0182648 PMC5546630

[CR50] Hummos A, Franklin CC, Nair SS (2014) Intrinsic mechanisms stabilize encoding and retrieval circuits differentially in a hippocampal network model. Hippocampus 24:1430–1448. 10.1002/HIPO.22324 24978936 10.1002/hipo.22324 PMC9121438

[CR51] Joshi A, Somogyi P (2020) Changing phase relationship of the stepping rhythm to neuronal oscillatory theta activity in the septo-hippocampal network of mice. Brain Struct Funct 225:871–879. 10.1007/s00429-020-02031-8 32060639 10.1007/s00429-020-02031-8 PMC7046600

[CR52] Joshi A, Salib M, Viney TJ et al (2017) Behavior-dependent activity and synaptic organization of septo-hippocampal GABAergic neurons selectively targeting the hippocampal CA3 area. Neuron 96:1342-1357.e5. 10.1016/j.neuron.2017.10.033 29198757 10.1016/j.neuron.2017.10.033 PMC5746169

[CR53] Karagiannis A, Gallopin T, Dávid C et al (2009) Classification of NPY-expressing neocortical interneurons. J Neurosci 29:3642. 10.1523/JNEUROSCI.0058-09.2009 19295167 10.1523/JNEUROSCI.0058-09.2009 PMC2750888

[CR54] Karalis N, Dejean C, Chaudun F et al (2016) 4-Hz oscillations synchronize prefrontal–amygdala circuits during fear behavior. Nat Neurosci 19:605–612. 10.1038/nn.4251 26878674 10.1038/nn.4251 PMC4843971

[CR55] Kawaguchi Y, Kubota Y (1996) Physiological and morphological identification of somatostatin- or vasoactive intestinal polypeptide-containing cells among GABAergic cell subtypes in rat frontal cortex. J Neurosci 16:2701. 10.1523/JNEUROSCI.16-08-02701.1996 8786446 10.1523/JNEUROSCI.16-08-02701.1996 PMC6578756

[CR56] Kellis DM, Kaigler KF, Witherspoon E et al (2020) Cholinergic neurotransmission in the basolateral amygdala during cued fear extinction. Neurobiol Stress 13:100279. 10.1016/j.ynstr.2020.100279 33344731 10.1016/j.ynstr.2020.100279 PMC7739185

[CR57] Kim D, Pare D, Nair SS (2013) Mechanisms contributing to the induction and storage of Pavlovian fear memories in the lateral amygdala. Learn Mem 20:421. 10.1101/LM.030262.113 23864645 10.1101/lm.030262.113 PMC3718199

[CR58] King C, Recce M, O’Keefe J (1998) The rhythmicity of cells of the medial septum/diagonal band of broca in the awake freely moving rat: relationships with behaviour and hippocampal theta. Eur J Neurosci 10:464–477. 10.1046/j.1460-9568.1998.00026.x 9749709 10.1046/j.1460-9568.1998.00026.x

[CR59] Kingir E, Sevinc C, Unal G (2023) Chronic oral ketamine prevents anhedonia and alters neuronal activation in the lateral habenula and nucleus accumbens in rats under chronic unpredictable mild stress. Neuropharmacology 228:109468. 10.1016/j.neuropharm.2023.109468 36813161 10.1016/j.neuropharm.2023.109468

[CR60] Király B, Domonkos A, Jelitai M et al (2023) The medial septum controls hippocampal supra-theta oscillations. Nat Commun 14:6159. 10.1038/s41467-023-41746-0 37816713 10.1038/s41467-023-41746-0 PMC10564782

[CR61] Knowland D, Lilascharoen V, Pacia CP, Shin S, Wang EH, Lim BK (2017) Distinct ventral pallidal neural populations mediate separate symptoms of depression. Cell 170(2):284–297.e18. 10.1016/j.cell.2017.06.015 28689640 10.1016/j.cell.2017.06.015 PMC5621481

[CR62] Kocsis B, Martínez-Bellver S, Fiáth R et al (2022) Huygens synchronization of medial septal pacemaker neurons generates hippocampal theta oscillation. Cell Rep 40:111149. 10.1016/j.celrep.2022.111149 35926456 10.1016/j.celrep.2022.111149

[CR63] Krabbe S, Gründemann J, Lüthi A (2018) Amygdala inhibitory circuits regulate associative fear conditioning. Biol Psychiatry 83:800–809. 10.1016/j.biopsych.2017.10.006 29174478 10.1016/j.biopsych.2017.10.006

[CR64] Lee S, Kim J-H (2019) Basal forebrain cholinergic-induced activation of cholecystokinin inhibitory neurons in the basolateral amygdala. Exp Neurobiol 28:320–328. 10.5607/en.2019.28.3.320 31308792 10.5607/en.2019.28.3.320 PMC6614066

[CR65] Lee MG, Chrobak JJ, Sik A et al (1994) Hippocampal theta activity following selective lesion of the septal cholinergic system. Neuroscience 62:1033–1047. 10.1016/0306-4522(94)90341-7 7845584 10.1016/0306-4522(94)90341-7

[CR66] Lesting J, Narayanan RT, Kluge C et al (2011) Patterns of coupled theta activity in amygdala-hippocampal-prefrontal cortical circuits during fear extinction. PLoS ONE 6:e21714 21738775 10.1371/journal.pone.0021714 PMC3125298

[CR67] Li G, Nair SS, Quirk GJ (2009) A biologically realistic network model of acquisition and extinction of conditioned fear associations in lateral amygdala neurons. J Neurophysiol 101:1629. 10.1152/JN.90765.2008 19036872 10.1152/jn.90765.2008 PMC2666411

[CR68] Lindén H, Hagen E, Łeski S et al (2014) LFPy: A tool for biophysical simulation of extracellular potentials generated by detailed model neurons. Front Neuroinform 7:41. 10.3389/FNINF.2013.00041/XML/NLM 24474916 10.3389/fninf.2013.00041 PMC3893572

[CR69] Lucas EK, Jegarl AM, Morishita H, Clem RL (2016) Multimodal and site-specific plasticity of amygdala parvalbumin interneurons after fear learning. Neuron 91:629–643. 10.1016/j.neuron.2016.06.032 27427462 10.1016/j.neuron.2016.06.032 PMC4975985

[CR70] Mahanty NK, Sah P (1998) Calcium-permeable AMPA receptors mediate long-term potentiation in interneurons in the amygdala. Nature 394:683–687. 10.1038/29312 9716132 10.1038/29312

[CR71] Mascagni F, McDonald AJ (2003) Immunohistochemical characterization of cholecystokinin containing neurons in the rat basolateral amygdala. Brain Res 976:171–184. 10.1016/S0006-8993(03)02625-8 12763251 10.1016/s0006-8993(03)02625-8

[CR72] Mascagni F, McDonald AJ (2009) Parvalbumin-immunoreactive neurons and GABAergic neurons of the basal forebrain project to the rat basolateral amygdala. Neuroscience 160:805. 10.1016/J.NEUROSCIENCE.2009.02.077 19285116 10.1016/j.neuroscience.2009.02.077 PMC2676771

[CR73] McDonald AJ (1997) Calbindin-D28k immunoreactivity in the rat amygdala. J Comp Neurol 383:231–244 9182851

[CR74] McDonald AJ (2020) Functional neuroanatomy of the basolateral amygdala: neurons, neurotransmitters, and circuits. Handb Behav Neurosci 26:1. 10.1016/B978-0-12-815134-1.00001-5 34220399 10.1016/b978-0-12-815134-1.00001-5 PMC8248694

[CR75] McDonald AJ, Mascagni F (2001) Colocalization of calcium-binding proteins and GABA in neurons of the rat basolateral amygdala. Neuroscience 105:681–693. 10.1016/S0306-4522(01)00214-7 11516833 10.1016/s0306-4522(01)00214-7

[CR76] McDonald AJ, Mascagni F (2010) Neuronal localization of m1 muscarinic receptor immunoreactivity in the rat basolateral amygdala. Brain Struct Funct 215:37–48. 10.1007/s00429-010-0272-y 20503057 10.1007/s00429-010-0272-y PMC4586030

[CR77] McDonald AJ, Mascagni F (2011) Neuronal localization of M2 muscarinic receptor immunoreactivity in the rat amygdala. Neuroscience 196:49–65. 10.1016/j.neuroscience.2011.08.032 21875654 10.1016/j.neuroscience.2011.08.032 PMC4586024

[CR78] McDonald AJ, Muller JF, Mascagni F (2011) Postsynaptic targets of GABAergic basal forebrain projections to the basolateral amygdala. Neuroscience 183:144–159. 10.1016/j.neuroscience.2011.03.027 21435381 10.1016/j.neuroscience.2011.03.027 PMC4586026

[CR79] McKenna JT, Yang C, Bellio T et al (2021) Characterization of basal forebrain glutamate neurons suggests a role in control of arousal and avoidance behavior. Brain Struct Funct 226:1755–1778. 10.1007/s00429-021-02288-7 33997911 10.1007/s00429-021-02288-7 PMC8340131

[CR80] McNaughton N, Ruan M, Woodnorth MA (2006) Restoring theta-like rythmicity in rats restores initial learning in the morris water maze. Hippocampus 16:1102–1110. 10.1002/hipo.20235 17068783 10.1002/hipo.20235

[CR81] Mesulam MM, Mufson EJ, Wainer BH, Levey AI (1983) Central cholinergic pathways in the rat: an overview based on an alternative nomenclature (Ch1–Ch6). Neuroscience 10:1185–1201. 10.1016/0306-4522(83)90108-2 6320048 10.1016/0306-4522(83)90108-2

[CR82] Minneci F, Janahmadi M, Migliore M et al (2007) Signaling properties of stratum oriens interneurons in the hippocampus of transgenic mice expressing EGFP in a subset of somatostatin-containing cells. Hippocampus 17:538–553. 10.1002/hipo.20291 17455332 10.1002/hipo.20291

[CR83] Mongia S, Tripathi A, Mengual E (2016) Arborization patterns of amygdalopetal axons from the rat ventral pallidum. Brain Struct Funct 221:4549–4573. 10.1007/s00429-016-1184-2 26832919 10.1007/s00429-016-1184-2

[CR84] Muller JF, Mascagni F, McDonald AJ (2011) Cholinergic innervation of pyramidal cells and parvalbumin-immunoreactive interneurons in the rat basolateral amygdala. J Comp Neurol 519:790. 10.1002/CNE.22550 21246555 10.1002/cne.22550 PMC4586025

[CR85] Mysin IE, Kitchigina VF, Kazanovich YB (2019) Phase relations of theta oscillations in a computer model of the hippocampal CA1 field: key role of Schaffer collaterals. Neural Netw 116:119–138. 10.1016/j.neunet.2019.04.004 31029053 10.1016/j.neunet.2019.04.004

[CR86] Nickerson Poulin A, Guerci A, El Mestikawy S, Semba K (2006) Vesicular glutamate transporter 3 immunoreactivity is present in cholinergic basal forebrain neurons projecting to the basolateral amygdala in rat. J Comp Neurol 498:690–711. 10.1002/cne.21081 16917846 10.1002/cne.21081

[CR87] O’Keefe J (1993) Hippocampus, theta, and spatial memory. Curr Opin Neurobiol 3:917–924. 10.1016/0959-4388(93)90163-S 8124075 10.1016/0959-4388(93)90163-s

[CR88] Ozawa M, Davis P, Ni J et al (2020) Experience-dependent resonance in amygdalo-cortical circuits supports fear memory retrieval following extinction. Nat Commun 11:4358. 10.1038/s41467-020-18199-w 32868768 10.1038/s41467-020-18199-w PMC7459312

[CR89] Pang KCH, Nocera R, Secor AJ, Yoder RM (2001) GABAergic septohippocampal neurons are not necessary for spatial memory. Hippocampus 11:814–827. 10.1002/HIPO.1097 11811676 10.1002/hipo.1097

[CR90] Pape H-C, Driesang RB (1998) Ionic mechanisms of intrinsic oscillations in neurons of the basolateral amygdaloid complex. J Neurophysiol 79:217–226. 10.1152/jn.1998.79.1.217 9425193 10.1152/jn.1998.79.1.217

[CR91] Pape H-C, Paré D, Driesang RB (1998) Two types of intrinsic oscillations in neurons of the lateral and basolateral nuclei of the amygdala. J Neurophysiol 79:205–216. 10.1152/jn.1998.79.1.205 9425192 10.1152/jn.1998.79.1.205

[CR92] Parasuram H, Nair B, D’Angelo E et al (2016) Computational modeling of single neuron extracellular electric potentials and network local field potentials using LFPsim. Front Comput Neurosci 10:65. 10.3389/FNCOM.2016.00065 27445781 10.3389/fncom.2016.00065 PMC4923190

[CR93] Paré D, Collins DR (2000) Neuronal correlates of fear in the lateral amygdala: multiple extracellular recordings in conscious cats. J Neurosci 20:2701. 10.1523/JNEUROSCI.20-07-02701.2000 10729351 10.1523/JNEUROSCI.20-07-02701.2000 PMC6772231

[CR94] Paré D, Collins DR, Pelletier JG (2002) Amygdala oscillations and the consolidation of emotional memories. Trends Cogn Sci 6:306–314. 10.1016/S1364-6613(02)01924-1 12110364 10.1016/s1364-6613(02)01924-1

[CR95] Paxinos G, Watson C (2007) The rat brain in stereotaxic coordinates, hard cover edition. Elsevier

[CR96] Porter JT, Cauli B, Staiger JF et al (1998) Properties of bipolar VIPergic interneurons and their excitation by pyramidal neurons in the rat neocortex. Eur J Neurosci 10:3617–3628. 10.1046/j.1460-9568.1998.00367.x 9875341 10.1046/j.1460-9568.1998.00367.x

[CR97] Power JM, Bocklisch C, Curby P, Sah P (2011) Location and function of the slow afterhyperpolarization channels in the basolateral amygdala. J Neurosci 31:526–537. 10.1523/JNEUROSCI.1045-10.2011 21228162 10.1523/JNEUROSCI.1045-10.2011 PMC6623441

[CR98] Rainnie DG, Mania I, Mascagni F, McDonald AJ (2006) Physiological and morphological characterization of parvalbumin-containing interneurons of the rat basolateral amygdala. J Comp Neurol 498:142–161. 10.1002/cne.21049 16856165 10.1002/cne.21049

[CR99] Rhomberg T, Rovira-Esteban L, Vikór A et al (2018) Vasoactive intestinal polypeptide-immunoreactive interneurons within circuits of the mouse basolateral amygdala. J Neurosci 38:6983. 10.1523/JNEUROSCI.2063-17.2018 29954847 10.1523/JNEUROSCI.2063-17.2018 PMC6070667

[CR100] Riedemann T (2019) Diversity and function of somatostatin-expressing interneurons in the cerebral cortex. Int J Mol Sci. 10.3390/ijms20122952 31212931 10.3390/ijms20122952 PMC6627222

[CR101] Roland JJ, Savage LM (2009) The role of cholinergic and GABAergic medial septal/diagonal band cell populations in the emergence of diencephalic amnesia. Neuroscience 160:32. 10.1016/J.NEUROSCIENCE.2009.02.044 19264109 10.1016/j.neuroscience.2009.02.044 PMC2899313

[CR102] Roland JJ, Stewart AL, Janke KL et al (2014) Medial septum-diagonal band of broca (MSDB) GABAergic regulation of hippocampal acetylcholine efflux Is dependent on cognitive demands. J Neurosci 34:506. 10.1523/JNEUROSCI.2352-13.2014 24403150 10.1523/JNEUROSCI.2352-13.2014 PMC3870934

[CR103] Sah P, Westbrook RF (2008) The circuit of fear. Nature 454:589–590. 10.1038/454589a 18668096 10.1038/454589a

[CR104] Sah P, Faber ESL, Lopez de Armentia M, Power J (2003) The amygdaloid complex: anatomy and physiology. Physiol Rev 83:803–834. 10.1152/physrev.00002.2003 12843409 10.1152/physrev.00002.2003

[CR105] Saunders A, Granger AJ, Sabatini BL (2015) Corelease of acetylcholine and GABA from cholinergic forebrain neurons. Elife 4:e06412. 10.7554/eLife.06412 25723967 10.7554/eLife.06412 PMC4371381

[CR106] Schindelin J, Arganda-Carreras I, Frise E et al (2012) Fiji: an open-source platform for biological-image analysis. Nat Methods 9:676–682. 10.1038/nmeth.2019 22743772 10.1038/nmeth.2019 PMC3855844

[CR107] Schlesiger MI, Ruff T, MacLaren DAA et al (2021) Two septal-entorhinal GABAergic projections differentially control coding properties of spatially tuned neurons in the medial entorhinal cortex. Cell Rep. 10.1016/j.celrep.2021.108801 33657367 10.1016/j.celrep.2021.108801

[CR108] Seidenbecher T, Laxmi TR, Stork O, Pape H-C (2003) Amygdalar and hippocampal theta rhythm synchronization during fear memory retrieval. Science 301:846–850. 10.1126/science.1085818 12907806 10.1126/science.1085818

[CR109] Shin LM, Liberzon I (2010) The neurocircuitry of fear, stress, and anxiety disorders. Neuropsychopharmacology 35:169–191. 10.1038/npp.2009.83 19625997 10.1038/npp.2009.83 PMC3055419

[CR110] Silberberg G, Markram H (2007) Disynaptic inhibition between neocortical pyramidal cells mediated by martinotti cells. Neuron 53:735–746. 10.1016/j.neuron.2007.02.012 17329212 10.1016/j.neuron.2007.02.012

[CR111] Silberberg G, Wu C, Markram H (2004) Synaptic dynamics control the timing of neuronal excitation in the activated neocortical microcircuit. J Physiol 556:19–27. 10.1113/jphysiol.2004.060962 14978208 10.1113/jphysiol.2004.060962 PMC1664894

[CR112] Skirzewski M, López W, Mosquera E et al (2011) Enhanced GABAergic tone in the ventral pallidum: memory of unpleasant experiences? Neuroscience 196:131–146. 10.1016/j.neuroscience.2011.08.058 21914462 10.1016/j.neuroscience.2011.08.058

[CR113] Smith Y, Paré J-F, Paré D (2000) Differential innervation of parvalbumin-immunoreactive interneurons of the basolateral amygdaloid complex by cortical and intrinsic inputs. J Comp Neurol 416:496–508 10660880

[CR114] Somogyi P, Katona L, Klausberger T et al (2014) Temporal redistribution of inhibition over neuronal subcellular domains underlies state-dependent rhythmic change of excitability in the hippocampus. Philos Trans R Soc Lond B Biol Sci 369:20120518. 10.1098/rstb.2012.0518 24366131 10.1098/rstb.2012.0518 PMC3866441

[CR115] Sosulina L, Graebenitz S, Pape H-C (2010) GABAergic interneurons in the mouse lateral amygdala: a classification study. J Neurophysiol 104:617–626. 10.1152/jn.00207.2010 20484532 10.1152/jn.00207.2010

[CR116] Stephenson-Jones M (2019) Pallidal circuits for aversive motivation and learning. Curr Opin Behav Sci 26:82–89. 10.1016/j.cobeha.2018.09.015

[CR117] Stephenson-Jones M, Bravo-Rivera C, Ahrens S et al (2020) Opposing contributions of GABAergic and glutamatergic ventral pallidal neurons to motivational behaviors. Neuron 105:921-933.e5. 10.1016/j.neuron.2019.12.006 31948733 10.1016/j.neuron.2019.12.006 PMC8573387

[CR118] Stujenske JM, Likhtik E, Topiwala MA, Gordon JA (2014) Fear and safety engage competing patterns of theta-gamma coupling in the basolateral amygdala. Neuron 83:919–933. 10.1016/j.neuron.2014.07.026 25144877 10.1016/j.neuron.2014.07.026 PMC4141236

[CR119] Takács VT, Cserép C, Schlingloff D et al (2018) Co-transmission of acetylcholine and GABA regulates hippocampal states. Nat Commun 9:2848. 10.1038/s41467-018-05136-1 30030438 10.1038/s41467-018-05136-1 PMC6054650

[CR120] Thomson AM, Deuchars J (1997) Synaptic interactions in neocortical local circuits: dual intracellular recordings in vitro. Cereb Cortex 7:510–522. 10.1093/cercor/7.6.510 9276176 10.1093/cercor/7.6.510

[CR121] Tóth K, Freund TF, Miles R (1997) Disinhibition of rat hippocampal pyramidal cells by GABAergic afferents from the septum. J Physiol 500:463–474. 10.1113/jphysiol.1997.sp022033 9147330 10.1113/jphysiol.1997.sp022033 PMC1159396

[CR122] Totty MS, Maren S (2022) Neural oscillations in aversively motivated behavior. Front Behav Neurosci 16:936036. 10.3389/fnbeh.2022.936036 35846784 10.3389/fnbeh.2022.936036 PMC9284508

[CR123] Totty MS, Tuna T, Ramanathan KR et al (2023) Thalamic nucleus reuniens coordinates prefrontal-hippocampal synchrony to suppress extinguished fear. Nat Commun 14:6565. 10.1038/s41467-023-42315-1 37848425 10.1038/s41467-023-42315-1 PMC10582091

[CR124] Unal CT, Pare D, Zaborszky L (2015a) Impact of basal forebrain cholinergic inputs on basolateral amygdala neurons. J Neurosci 35:853. 10.1523/JNEUROSCI.2706-14.2015 25589777 10.1523/JNEUROSCI.2706-14.2015 PMC4293427

[CR125] Unal G, Joshi A, Viney TJ et al (2015b) Synaptic targets of medial septal projections in the hippocampus and extrahippocampal cortices of the mouse. J Neurosci 35:15812–15826. 10.1523/JNEUROSCI.2639-15.2015 26631464 10.1523/JNEUROSCI.2639-15.2015 PMC4666911

[CR126] Unal G, Crump MG, Viney TJ et al (2018) Spatio-temporal specialization of GABAergic septo-hippocampal neurons for rhythmic network activity. Brain Struct Funct 223:2409–2432. 10.1007/s00429-018-1626-0 29500537 10.1007/s00429-018-1626-0 PMC5968071

[CR127] Vandecasteele M, Varga V, Berényi A et al (2014) Optogenetic activation of septal cholinergic neurons suppresses sharp wave ripples and enhances theta oscillations in the hippocampus. Proc Natl Acad Sci U S A 111:13535–13540. 10.1073/pnas.1411233111 25197052 10.1073/pnas.1411233111 PMC4169920

[CR128] Varga V, Hangya B, Kránitz K et al (2008) The presence of pacemaker HCN channels identifies theta rhythmic GABAergic neurons in the medial septum. J Physiol 586:3893–3915. 10.1113/jphysiol.2008.155242 18565991 10.1113/jphysiol.2008.155242 PMC2538919

[CR129] Vega-Flores G, Rubio SE, Jurado-Parras MT et al (2014) The GABAergic septohippocampal pathway is directly involved in internal processes related to operant reward learning. Cereb Cortex 24:2093. 10.1093/CERCOR/BHT060 23479403 10.1093/cercor/bht060 PMC4441070

[CR130] Washburn MS, Moises HC (1992) Electrophysiological and morphological properties of rat basolateral amygdaloid neurons in vitro. J Neurosci 12:4066–4079. 10.1523/JNEUROSCI.12-10-04066.1992 1403101 10.1523/JNEUROSCI.12-10-04066.1992 PMC6575963

[CR131] Weisskopf MG, Bauer EP, LeDoux JE (1999) L-type voltage-gated calcium channels mediate NMDA-independent associative long-term potentiation at thalamic input synapses to the amygdala. J Neurosci 19:10512 LP – 10519. 10.1523/JNEUROSCI.19-23-10512.1999 10575047 10.1523/JNEUROSCI.19-23-10512.1999 PMC6782436

[CR132] Woodruff AR, Sah P (2007) Inhibition and synchronization of basal amygdala principal neuron spiking by parvalbumin-positive interneurons. J Neurophysiol 98:2956–2961. 10.1152/jn.00739.2007 17715201 10.1152/jn.00739.2007

[CR133] Woolf NJ, Butcher LL (1982) Cholinergic projections to the basolateral amygdala: a combined evans blue and acetylcholinesterase analysis. Brain Res Bull 8:751–763. 10.1016/0361-9230(82)90102-2 6182963 10.1016/0361-9230(82)90102-2

[CR134] Wulff P, Ponomarenko AA, Bartos M et al (2009) Hippocampal theta rhythm and its coupling with gamma oscillations require fast inhibition onto parvalbumin-positive interneurons. Proc Natl Acad Sci U S A 106:3561–3566. 10.1073/pnas.0813176106 19204281 10.1073/pnas.0813176106 PMC2637907

[CR135] Xu C, Datta S, Wu M, Alreja M (2004) Hippocampal theta rhythm is reduced by suppression of the H-current in septohippocampal GABAergic neurons. Eur J Neurosci 19:2299–2309. 10.1111/J.0953-816X.2004.03316.X 15090056 10.1111/j.0953-816X.2004.03316.x

[CR136] Yau JO-Y, Chaichim C, Power JM, McNally GP (2021) The roles of basolateral amygdala parvalbumin neurons in fear learning. J Neurosci 41:9223. 10.1523/JNEUROSCI.2461-20.2021 34561234 10.1523/JNEUROSCI.2461-20.2021 PMC8570827

[CR137] Yoder RM, Pang KCH (2005) Involvement of GABAergic and cholinergic medial septal neurons in hippocampal theta rhythm. Hippocampus 15:381–392. 10.1002/HIPO.20062 15630696 10.1002/hipo.20062

[CR138] Zaborszky L, Pang K, Somogyi J et al (1999) The basal forebrain corticopetal system revisited. Ann N Y Acad Sci 877:339–367. 10.1111/j.1749-6632.1999.tb09276.x 10415658 10.1111/j.1749-6632.1999.tb09276.x

[CR139] Zaborszky L, Csordas A, Mosca K et al (2015) Neurons in the basal forebrain project to the cortex in a complex topographic organization that reflects corticocortical connectivity patterns: an experimental study based on retrograde tracing and 3D reconstruction. Cereb Cortex 25:118–137. 10.1093/cercor/bht210 23964066 10.1093/cercor/bht210 PMC4259277

[CR140] Zador A, Koch C, Brown TH (1990) Biophysical model of a Hebbian synapse. Proc Natl Acad Sci U S A 87:6718. 10.1073/PNAS.87.17.6718 2168555 10.1073/pnas.87.17.6718 PMC54608

[CR141] Zingg B, Dong H-W, Tao HW, Zhang LI (2022) Application of AAV1 for anterograde transsynaptic circuit mapping and input-dependent neuronal cataloging. Curr Protoc 2:e339. 10.1002/cpz1.339 35044725 10.1002/cpz1.339 PMC8852298

